# Less favourable climates constrain demographic strategies in plants

**DOI:** 10.1111/ele.12794

**Published:** 2017-06-13

**Authors:** Anna M. Csergő, Roberto Salguero‐Gómez, Olivier Broennimann, Shaun R. Coutts, Antoine Guisan, Amy L. Angert, Erik Welk, Iain Stott, Brian J. Enquist, Brian McGill, Jens‐Christian Svenning, Cyrille Violle, Yvonne M. Buckley

**Affiliations:** ^1^ Department of Zoology School of Natural Sciences Trinity College Dublin College Green Dublin 2 Ireland; ^2^ ARC Centre of Excellence for Environmental Decisions University of Queensland St Lucia QLD 4072 Australia; ^3^ Department of Animal and Plant Sciences University of Sheffield Western Bank Sheffield S10 2TN UK; ^4^ Max Planck Institute for Demographic Research Konrad‐Zuse‐Straße 1 18057 Rostock Germany; ^5^ Department of Ecology and Evolution University of Lausanne 1015 Lausanne Switzerland; ^6^ Institute of Earth Surface Dynamics University of Lausanne 1015 Lausanne Switzerland; ^7^ Departments of Botany and Zoology Biodiversity Research Centre University of British Columbia 115‐2212 Main Mall Vancouver BC Canada; ^8^ Institute of Biology Martin‐Luther‐University Halle‐Wittenberg 06099 Halle (Saale) Germany; ^9^ German Centre for Integrative Biodiversity Research (iDiv) Halle‐Jena‐Leipzig 04103 Leipzig Germany; ^10^ Department of Biology Max Planck Odense Center University of Southern Denmark Campusvej 55 DK‐5230 Odense M Denmark; ^11^ Department of Ecology and Evolutionary Biology The University of Arizona 1040 E. Lowell St. Tucson AZ 85721 USA; ^12^ School of Biology and Ecology The University of Maine Deering Hall Room 202 Orono ME 04469 USA; ^13^ Section for Ecoinformatics & Biodiversity Department of Bioscience Aarhus University Ny Munkegade 114 DK‐8000 Aarhus C Denmark; ^14^ CNRS, CEFE UMR 5175, Université de Montpellier – Université Paul Valéry – EPHE 1919 Route de Mende 34293 Montpellier Cedex 5 France

**Keywords:** Climate change, COMPADRE Plant Matrix Database, demographic compensation, ecological niche models, matrix population models, population dynamics, spatial demography, species distribution models, species interactions–abiotic stress hypothesis, stress gradient hypothesis

## Abstract

Correlative species distribution models are based on the observed relationship between species’ occurrence and macroclimate or other environmental variables. In climates predicted less favourable populations are expected to decline, and in favourable climates they are expected to persist. However, little comparative empirical support exists for a relationship between predicted climate suitability and population performance. We found that the performance of 93 populations of 34 plant species worldwide – as measured by *in situ* population growth rate, its temporal variation and extinction risk – was not correlated with climate suitability. However, correlations of demographic processes underpinning population performance with climate suitability indicated both resistance and vulnerability pathways of population responses to climate: in less suitable climates, plants experienced greater retrogression (resistance pathway) and greater variability in some demographic rates (vulnerability pathway). While a range of demographic strategies occur within species’ climatic niches, demographic strategies are more constrained in climates predicted to be less suitable.

## Introduction

Contemporary climate change is causing rapid redistribution of species’ geographic ranges (Chen *et al*. 2011). Understanding how the environment affects population performance is key for predicting the response of different species to climate change and for designing conservation management strategies (Sutherland *et al*. 2013). Broad‐scale predictions of species’ geographic redistributions have largely relied on correlative species distribution models (SDMs), or ecological niche models, that predict habitat suitability from species presences and current macroclimate (e.g. Guisan & Zimmermann 2000; Guisan *et al*. 2013, S1; Mod *et al*. 2016). When projecting species’ range shifts, SDMs implicitly assume that current linkages between climate suitability and occurrence will hold in the future, which implies a functional link between macroclimate and demography (detailed below). While SDMs based purely on species’ presence may correctly predict patterns of local abundances (VanDerWal *et al*. 2009), their ability to predict the demographic processes that control species’ persistence remains uncertain (Thuiller *et al*. 2014).

**Figure 1:**
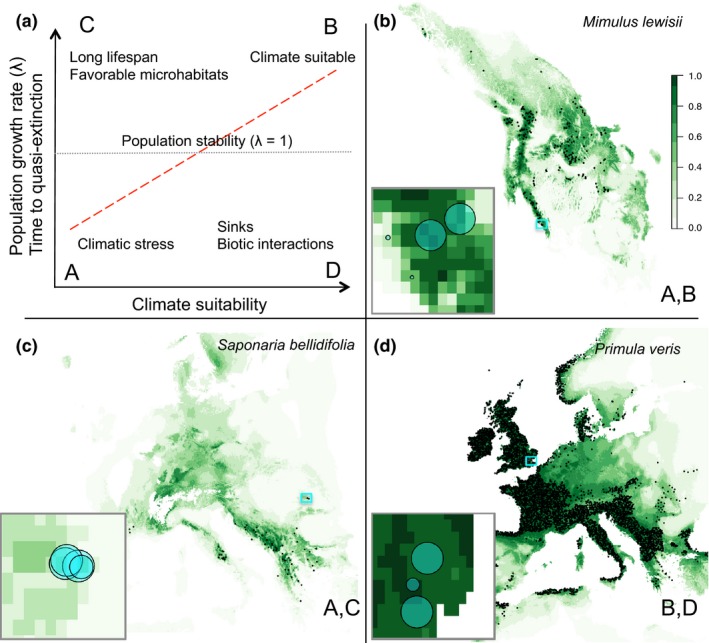
Theoretical expectations and example data showing potential relationships between climate suitability and population growth rate and extinction risk (time to quasi‐extinction). Climate suitability is from species distribution models based on species presences and macroclimate data and time to quasi‐extinction is estimated from demographic data for geo‐located populations in the COMPADRE Plant Matrix Database. Panel (a) represents a match‐mismatch chart showing possible relationships between climate suitability and population growth rate/time to quasi‐extinction: positive relationship (red dashed line through quadrants A and B) and deviations from this expectation due to different ecological processes (quadrants C and D**)**. The horizontal grey dotted line represents populations that neither increase nor decrease. Panels (b–d) show predicted climate suitability maps for three selected species in COMPADRE Plant Matrix Database, in line with the range of outcomes in panel (a) through quadrants A–D. Presences used to generate the projected climate suitability maps are represented as small black dots, and climate suitability values range from unsuitable in light green to highly suitable in dark green as indicated by the scale. Inserts are expanded climate suitability maps from the turquoise squares in the larger maps; centres of turquoise circles show the locations of COMPADRE populations and circle sizes are proportional to the value of the predicted time to quasi‐extinction (34–300 years)**.**

If macroclimates affect population processes, we may expect stable or increasing populations with low extinction risk in suitable macroclimates, and declining populations with high extinction risk in unsuitable macroclimates (Pulliam 2000) (Fig. 1a(A,B)). However, multiple ecological processes can decouple local population dynamics from macroclimate suitability (Fig. 1a(C,D); Pearson & Dawson 2003; Holt 2009). In suitable climates, populations may decline due to intense biotic interactions (Holt 2009; Louthan *et al*. 2015) (Fig. 1a(D)). In unsuitable climates, populations may persist temporarily under apparent demographic balance if confined to favourable microhabitats, through demographic buffering, or because the time scales experienced by organisms differ from the time scale of climate change (e.g. long‐lived relict populations; Dullinger *et al*. 2012; Hylander & Ehrlén 2013) (Fig. 1a(C) and ‘Population stability’ (λ* *= 1)). Rapid population increase may be expected across a range of climatic suitabilities: in suitable climates when populations regenerate after disturbances (Holt 2009) and in unsuitable climates in occasional favourable years (Fig. 1a(B,C)). Even where populations are already established, transient (short‐term) perturbations to demographic rates and consequent effects on population structure may affect population dynamics independently of climate (Stott *et al*. 2011). Consequently, mismatches between macroclimate suitability and observed population performance may be common (Thuiller *et al*. 2014).

Even if key demographic processes such as fecundity and survival vary substantially across climatic gradients, their integrated effects on overall population performance may remain low. Compensatory demographic responses to environmental changes may maintain stable (i.e. neither declining or increasing) populations over different environments and across large spatial extents (Villellas *et al*. 2015; Tavecchia *et al*. 2016; Treurnicht *et al*. 2016). Natural selection can shape life‐history strategies, buffering populations from fitness declines across the range of experienced climatic variation (Pfister 1998). Negative density‐dependent processes (e.g. competition and diseases) in suitable climates and positive density‐dependent processes (e.g. Allee effect) in unsuitable climates may ultimately result in stable populations (Haldane 1956). Thus, the relationship between climate suitability and population growth rate (λ) and extinction risk might be weak over the range of climate suitabilities observed for a given species’ populations (Fig. 1) (Thuiller *et al*. 2014). Despite these limitations, dismissing the capabilities of correlative SDMs based on lack of a direct relationship with integrated metrics of population performance such as population growth rate could be erroneous. Evidence of climatic constraints on demographic processes that underlie the integrated measures of population performance (e.g. fecundity) may represent early warning signals of population collapse (Doak & Morris 2010). Consequently, determining the demographic pathways of population responses to the environment may be necessary to link predicted climate suitability to population performance, but to date this approach has not been taken in global comparative studies.

Data to test whether predictions of SDMs correlate with population performance have been scarce (Schurr *et al*. 2012; Guisan 2014; Ehrlén & Morris 2015). Spatially and temporally replicated demographic datasets across large geographic scales and environmental gradients have been lacking (Buckley *et al*. 2010). The poor resolution of demographic data on which existing tests relied have not allowed for estimation of demographic processes underlying overall population performance (Thuiller *et al*. 2014). The poor spatial replication of sites involved in comparative analyses limited the range of environmental conditions in which population were located to temperate regions, and the range of life forms studied mostly to tree species (Thuiller *et al*. 2014). Here, we capitalized on detailed demographic data for 34 tree and herbaceous perennial plant species from the COMPADRE Plant Matrix Database (Salguero‐Gómez *et al*. 2015), which were studied in a range of environments from temperate to tropical. We built presence‐only SDMs for these species and determined whether overall population performance and underlying demographic processes correlated with predictions of macroclimate suitability.

**Figure 2:**
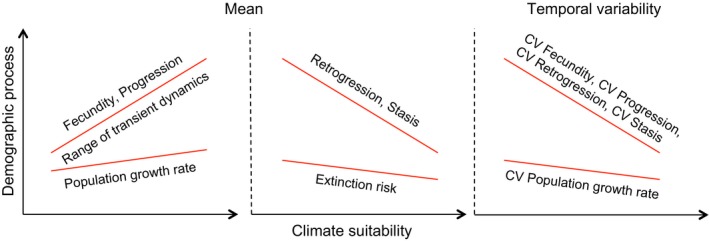
Naïve expectations of relationships between predicted climate suitability and mean and temporal variability of integrated population performance metrics and underlying demographic processes. We expected a positive relationship between climate suitability and population growth rate and negative relationships between climate suitability and extinction risk and the temporal variation (CV) in population growth rate. We expected the range of transient dynamics to increase with climate suitability. We expected positive relationships between climate suitability and the underlying demographic processes of fecundity and progression and negative relationships between climate suitability and the underlying processes of retrogression and stasis and the temporal variation (CV) in all basic demographic processes. If limits imposed by climate suitability on basic demographic processes are not fully integrated through the demographic performance, one outcome could be stronger relationships of climate suitability with basic demographic processes (fecundity, progression, retrogression, stasis) than with overall population performance metrics (population growth rate, extinction risk). CV = coefficient of variation across annual censuses, i.e. temporal variability in demographic performance.

We hypothesized that limits imposed by climate suitability on demographic processes are integrated through to overall population performance. Based on this naïve assumption, we expected a positive relationship between climate suitability and population growth rate, and a negative relationship between climate suitability and the temporal variation in the observed population growth rates and population extinction risk (Fig. 2). We further hypothesized that populations in more suitable climates can potentially recover more rapidly from lower population densities caused by local disturbances (e.g. local fires) and can reach higher population densities than populations in less suitable climates, where recovery may be slower and of lower magnitude (range of transient dynamics, Fig. 2).

We then tested whether predicted climate suitability was related to the demographic rates that underpin the integrated population performance: fecundity, progression (growth conditional on survival), retrogression (regression in size conditional on survival) and stasis (survival with no growth or regression). We predicted that low climate suitability would be associated with lower values of mean fecundity and progression and higher values of mean stasis and retrogression, and with more variable demographic rates (Fig. 2).

In addition, to determine whether climate suitability changes the relative importance of demographic processes for the population growth rate (λ), we tested whether elasticities of population growth rate to changes in demographic rates were correlated with climate suitability. Finally, to determine whether climate constraints on demographic rates might expose populations to extinction risk, we tested for direct relationships between mean and temporal variation in demographic rates and projected time to population quasi‐extinction.

## Methods

### Overview

We used multi‐year demographic data from the global COMPADRE Plant Matrix Database (Salguero‐Gómez *et al*. 2015, http://www.compadre-db.org; hereafter COMPADRE) for 93 populations across 34 species of trees and herbaceous perennials worldwide. We fitted presence‐based correlative SDMs from which we extracted macroclimate suitability (hereafter, climate suitability) values for the location of each population. We used linear mixed‐effects models (LMMs) to predict integrated population performance and underlying demographic processes using climate suitability as the primary predictor of interest.

### Demographic models

#### Data selection

We extracted matrix projection models (MPMs) from COMPADRE 3.0.0, accessed on 21 July 2014. To achieve high demographic data quality, we imposed rigorous *a priori* study selection criteria (detailed in Appendix S1.1), resulting in an initial set of 123 species/311 populations. Of these, 34 species with 93 populations had presence data adequate for fitting quality SDMs, resulting in our final dataset.

#### Demographic metrics

##### Mean and variation in population growth rate (λ)

Multiple deterministic and stochastic measures of λ are typically used to describe population growth rate (Buckley *et al*. 2010). The five mean λ metrics examined here were highly correlated (details in Appendix S1.2), therefore, we present only results for the stochastic population growth rate in an independent and identically distributed environment (λ_*iid*_). Temporal variation in λ for each population was calculated as the coefficient of variation in deterministic growth rates (CV_λdet_) from annual MPMs for each population.

##### Time to quasi‐extinction

Because λ values only assess whether populations are likely to grow, stay unchanged or decline, but not the extinction risk *per se* (Caswell 2001), we estimated the time to local population extinction by simulating population densities over time with stochastic quasi‐extinction probability curves (Caswell 2001). Using initial population sizes of 200 individuals/population (chosen as detailed in Appendix S1.2), we calculated the probability that above‐ground population size falls below a quasi‐extinction threshold of one individual (quasi‐extinction is defined as 95% probability of reaching a set threshold). Simulations were run over 300 years.

##### Transient population dynamics

Population performance can respond to environmental changes at different time scales. Under relatively stable environmental conditions long‐term estimates (asymptotic population dynamics) are of interest (Caswell 2001). For population responses to strong, short‐lived environmental perturbations (e.g. fire, disease epidemics, extreme weather events), non‐asymptotic (i.e. transient) dynamics over short‐ and longer‐term time frames are important. To estimate the response of populations to potential local disturbances across a range of climate suitabilities, we calculated two measures of transient dynamics: the reactivity range and the inertia range (Stott *et al*. 2011). High values of reactivity and inertia range indicate a wide range of potential population sizes immediately and over the long‐term, respectively, following a disturbance, thus gauging potential population responses to perturbations over two different time windows (details in Appendix S1.2).

##### Mean and variation in basic demographic processes

We extracted four fundamental demographic processes from each annual MPM for each population: fecundity, progression, retrogression and stasis between life stages, by calculating an average of matrix elements corresponding to each of these demographic transitions, weighted by the stable‐stage distribution (details in Appendix S1.2). For each species we calculated the arithmetic mean and coefficient of variation in all these demographic quantities over the study period (3.0 years ± 1.3 SD across studies).

##### The elasticity of population growth rate (λ) to changes in demographic transitions

To determine whether climate suitability changes the relative importance of demographic rates for λ, we calculated the elasticity of deterministic λ to changes in fecundity, progression, retrogression and stasis separately, by summing up the elasticity matrix entries corresponding to each of these demographic transitions in the mean MPM (Silvertown & Franco 1993).

Matrix projection models were analysed following standard procedures (Caswell 2001) using the *popbio* and *popdemo* packages (Stubben & Milligan 2007; Stott *et al*. 2012), and specific scripts developed in R 3.2.4 for these purposes. The general demographic characteristics of the studied populations are presented in Appendix S2.1.

### Species distribution models

#### Data selection

We compiled a dataset of species occurrences from multiple biodiversity repositories: GBIF (Global Biodiversity Information Facility), BIEN (Botanical Information and Ecology Network), local and regional herbaria and digitized species distribution maps from atlases (Appendix S1.1). We checked the occurrence dataset for coordinate accuracy and precision and removed cultivated specimens, non‐native occurrences and duplicated records, and then performed spatial resampling as described in Appendix S1.1 to avoid spatial autocorrelation. We selected a set of eight climatic predictors commonly used in distribution models for plant species, downloaded at 5 arc‐min resolution (~ 10 km in temperate regions) to model climatic niches: annual mean temperature, temperature seasonality, mean temperature of warmest quarter, mean temperature of coldest quarter, precipitation seasonality and precipitation of wettest quarter extracted from the WORLDCLIM dataset (Hijmans *et al*. 2005) and annual and seasonality of global potential evapotranspiration, extracted from the CGIAR Consortium for Spatial Information (Appendix S1.3).

#### Modelling approach

As SDM predictions are sensitive to different computational approaches (Elith *et al*. 2006), we used an ensemble of four different techniques to obtain robust estimates: generalized linear models, generalized boosted regression models, random forest and maximum entropy modelling as implemented in the BIOMOD 2 library (Thuiller *et al*. 2009) (full details including model evaluation procedures are presented in Appendix S1.3). Because we used background points instead of true absence data and climate suitability values were not real occurrence probabilities, to make predictions comparable across species we rescaled the predicted climate suitability values to range between 0 and 1 with the following formula: (*x − min*)/(*max − min*). Thus, climate suitability of each grid cell was relative to the maximum climate suitability value observed for each species. Following this standardization, we extracted climate suitability values for the 10 × 10 km^2^ grid cells corresponding to the location of each COMPADRE population, a scale considered appropriate to model macroclimate suitability while also tolerating uncertainties in reported geographic coordinates in COMPADRE (Salguero‐Gómez *et al*. 2015).

### Statistical analyses

We fitted linear mixed‐effects models (LMMs) to examine the effect of climate suitability on mean and temporal variation in population growth rates, population extinction risk and the two measures of transient dynamics. Climate suitability, growth form (herbaceous perennial or tree) and two terms associated with MPM structure: matrix dimension (number of stages in life cycle) and study duration (number of projection matrices over the course of the study), known to affect demographic metrics (Salguero‐Gómez & Plotkin 2010; Crone *et al*. 2013) were modelled as fixed effects. The interaction between climate suitability and the rest of the terms was also specified with the exception of models of extinction risk, where no interactions were specified because of limited sample size (models were fit for *N* = 31 populations with quasi‐extinction time < 300 years). A random intercept by species was modelled to account for multiple populations within a species. More complex random effects by species (e.g. random slopes) were not modelled due to lack of power. As we used both within‐species and between‐species data to estimate the effect of climate suitability on population performance, we were not constrained to the range of suitability values encountered within a single species for inference. Because residual variance in models of population growth rate and temporal variation in population growth rate were observed to increase with increasing climate suitability (see Results), we compared models specified with both normal and gamma distribution of errors.

We fitted LMMs to explore the demographic pathways of the climate suitability effect on λ and extinction risk. We modelled the effect of climate suitability on the mean and temporal variation in fecundity, progression, retrogression and stasis, and on the elasticity of λ to changes in the mean of these demographic processes. Models were fitted in a similar way to those of λ above with few exceptions (see Appendix S2.3). In particular, models of elasticities contained two additional fixed terms: the stochastic population growth rate (λ_*iid*_) and the interaction between λ_*iid*_ and climate suitability introduced to control for the known correlation between elasticities and λ_*iid*_.

To explore the response of extinction risk to underlying demographic processes, with two separate models we tested the effects of mean and temporal variation in the demographic processes on population time to quasi‐extinction. In these models, fixed effects were matrix dimension, study length and either the mean or the temporal variation in fecundity, stasis, retrogression and progression. Random effects were specified in a similar way to the models of λ above.

To make the effect size of variables selected in the final model comparable, all continuous predictor variables were centered on 0 and scaled to have unit variance. Variables were log‐ or square root transformed if the transformation improved the distribution of residuals.

We used information‐theoretic multimodel inference (MuMIn package, Bartoń 2014) for model selection. This approach evaluates all possible variable combinations as nested subsets of the full model and ranks alternative models using the low sample size‐corrected version Akaike's information criterion (AICc), which penalizes models for extra parameters, limiting model overfitting. We opted for this exploratory analytical choice despite having *a priori* expectations about the relationship between population performance and climate suitability because the interaction of matrix dimension, study length and growth form with climate suitability has not been tested before and we lacked strong *a priori* expectations about these interactions. Models with the lowest AIC_c_ were selected to infer our results. Marginal *R* ^2^ values were calculated to describe the proportion of variance explained by the fixed factor(s).

Models were fitted with the *nlme* package (Pinheiro *et al*. 2014) in R 3.2.4 except for models fitted with gamma error distribution, for which we used the *lme4* package (Bates *et al*. 2014). Model details are provided in Appendix S2.3 in Supporting Information.

## Results

### The relationship between climate suitability and population growth rate, extinction risk and transient dynamics

**Figure 3:**
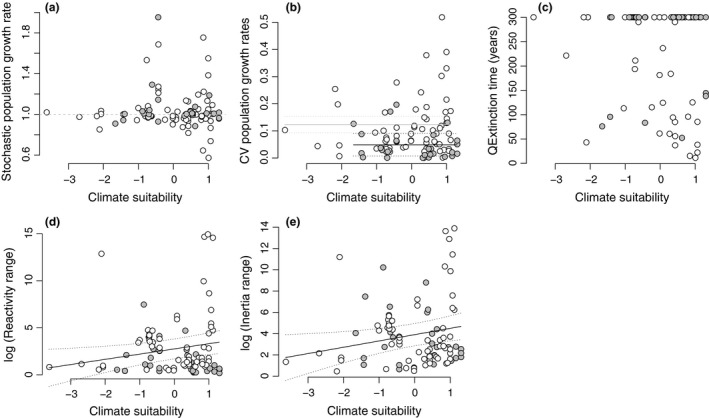
The relationship between climate suitability and integrated population performance: (a) stochastic population growth rates, (b) temporal variation of deterministic population growth rates and (c) time to quasi‐extinction and population transient dynamics: reactivity range (d) and inertia range (e) for 93 populations across 34 species of trees and herbaceous perennials. The linear mixed‐effects models revealed no relationships between climate suitability and stochastic population growth rates, temporal variation in population growth rates and time to quasi‐extinction, but showed an increase in the variance of population growth rate among populations and the temporal variation in population growth rates with climate suitability (model details are presented in Appendix S2.3). The two transient dynamic metrics were positively correlated with climate suitability (model details are presented in Table 1; Effect sizes are comparatively presented in Fig. 4). Trees are represented with grey filled circles, herbaceous perennials with empty circles. The dashed line in panel (a) represents stable, neither increasing nor declining populations (λ_*iid*_ = 1). Dotted lines represent 95% confidence intervals around the mean. CV = coefficient of variation across annual censuses, i.e. temporal variability in demographic performance. Climate suitability values are centered on zero with unit variance.

The examined populations tended to occur at the high end of the climate suitability gradient (mean observed climate suitability was 0.765 ± 0.178 SD; minimum climate suitability was 0.121, i.e. 12% of the maximum climate suitability for that species; Fig. 3a). However, populations with λ* *≥ 1 were also observed at low climate suitability values. The mean λ across all populations was near 1 (λ_*iid*_ = 1.04 ± 0.20 SD; Fig. 3a, for individual species see Appendix S2.2a), meaning that on average populations were neither increasing nor decreasing. There was no evidence that mean λ was related to climate suitability in either trees or herbaceous perennials (Fig. 3a, Appendix S2.3).

Herbs had a higher temporal variation in λ (CV_λdet_) than trees, but CV_λdet_ was not associated with climate suitability (Fig. 3b, Appendix S2.3). Models of both λ_*iid*_ and CV_λdet_ fitted with gamma distribution errors (to model the observed increase in residual values with the mean) had much stronger support than models with normal errors (AICc_gamma_ = −64.2 and AICc_normal_ = 13.1 and AICc_gamma_ = −257.3 and AICc_normal_ = −124.0, respectively), supporting a wider range of λ values and their temporal variation in more suitable climates (Fig. 3a,b).

The time to population quasi‐extinction was relatively high across all climate suitability values, but we found several populations at risk even in relatively suitable climates (Fig. 3c; for individual species see Appendix S2.2b). A positive relationship of climate suitability with time to quasi‐extinction was not supported in either trees or herbaceous perennials (Fig. 3c, Appendix S2.3). Figure 1 shows examples of individual species in which the prediction of a positive relationship between climate suitability and population time to quasi‐extinction was either met (panel (b)) or rejected (panels (c) and (d)).

**Figure 4:**
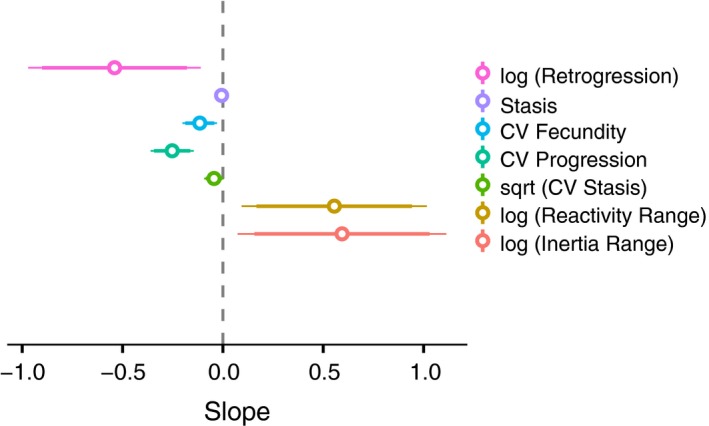
The effect size (slope and 95% confidence intervals) of climate suitability on mean retrogression and stasis, temporal variation in fecundity, progression and stasis and transient population dynamics (reactivity range and inertia range), modelled using linear mixed‐effects models. Positive slope values indicate a positive relationship between climate suitability and each response variable and negative values indicate a decline in response variables with climate suitability. CV = coefficient of variation across annual censuses. Model details are reported in Table 1.

The reactivity range and inertia range of populations increased with increasing climate suitability, indicating that following disturbances to population structure, persisting populations may experience larger changes in population size in more suitable climates – both immediately and over a longer term – compared to populations in less suitable climates (Figs 3d,e and 4, Table 1, Appendix S2.3).

**Table 1:** Best fit linear mixed‐effects models (LMMs) for the effects of climate suitability on population performance

Model structure and predicted variable	Selected variable	β	SE(β)	*R* ^2^
*Effects of climate suitability on transient dynamics*				
log(RR) ~ MD + SL + GT + CS + MD : CS + SL : CS + GT : CS				
Reactivity range	Intercept	2.751	0.541	0.029
	CS	0.555	0.235	
log(IR) ~ MD + SL + GT + CS + MD : CS + SL : CS + GT : CS				
Inertia range	Intercept	3.907	0.535	0.033
	CS	0.593	0.265	
*Effects of climate suitability on mean and temporal variation in demographic processes*				
stasis ~ MD + SL + CS + MD : CS + SL : CS				
Stasis	Intercept	0.100	0.009	0.531
	GT Tree	0.062	0.016	
	CS	−0.007	0.005	
	MD	0.041	0.007	
log(retr) ~ MD + SL + CS + MD : CS + SL : CS				
Retrogression (Herbaceous perennials)	Intercept	−6.312	0.614	0.037
	CS	−0.540	0.219	
Sqrt(CV_stasis) ~ MD + SL + GT + CS + MD : CS + SL : CS + GT : CS				
Temporal variation in stasis	Intercept	0.437	0.038	0.090
	GT Tree	−0.102	0.063	
	CS	−0.044	0.025	
CV_fec ~ MD + SL + GT + CS + MD : CS + SL : CS + GT : CS				
Temporal variation in fecundity	Intercept	0.510	0.048	0.086
	CS	−0.116	0.044	
CV_prog ~ MD + SL + CS				
Temporal variation in progression (Trees)	Intercept	0.488	0.050	0.486
	CS	−0.253	0.055	
	MD	−0.088	0.044	
*Effects of climate suitability on the elasticity of population growth rate to changes in demographic processes*				
ElastFec ~ MD + SL + GT + CS + λ_*iid*_ + MD : CS + SL : CS + GT : CS + λ_*iid*_ : CS				
Elasticity to fecundity	Intercept	0.077	0.011	0.401
	GT Tree	−0.042	0.018	
	CS	−0.018	0.005	
	λ_*iid*_	0.032	0.003	
	SL	−0.004	0.005	
	GT Tree : CS	0.029	0.011	
	λ_*iid*_ * *: CS	0.012	0.004	
	SL : CS	0.016	0.006	
ElastProg ~ MD + SL + GT + CS + λ_*iid*_ + MD : CS + SL : CS + GT : CS + λ_*iid*_ : CS				
Elasticity to progression	Intercept	0.251	0.021	0.506
	GT Tree	−0.138	0.035	
	CS	−0.007	0.013	
	λ_*iid*_	0.066	0.007	
	MD	0.042	0.016	
	MD : CS	0.023	0.013	
ElastStasis ~ MD + SL + GT + CS + λ_*iid*_ + MD : CS + SL : CS + GT : CS + λ_*iid*_ : CS				
Elasticity to stasis	Intercept	0.564	0.033	0.420
	GT Tree	0.292	0.054	
	CS	0.015	0.019	
	λ_*iid*_	−0.082	0.010	
	MD	−0.062	0.024	
	MD : CS	−0.037	0.020	
ElastRetr ~ MD + SL + GT + CS + λ_*iid*_ + MD : CS + SL : CS + GT : CS + λ_*iid*_ : CS				
Elasticity to retrogression	Intercept	0.092	0.013	0.320
	GT Tree	−0.064	0.031	
	CS	−0.005	0.006	
	λ_*iid*_	−0.030	0.005	
	SL	0.008	0.007	
	CS : SL	−0.020	0.008	

The first column shows the fixed effects in the full models and the abbreviated and full name of predicted variables. The next columns show the coefficient means β and standard errors SE(β) for variables selected in the best model, and marginal (fixed effects) *R* ^2^ values of the best models. In all models species (‘SpeciesAccepted’ column in COMPADRE) were introduced as random effects (intercept only). MD = matrix dimension, SL = study length, GT = growth type, CS = climate suitability, CV = coefficient of variation. Effect sizes for models of transient dynamics and underlying demographic processes are comparatively presented in Fig. 4. The results of elasticity models are graphically presented in Appendix S2.4. Models where climate suitability was not selected during model inference are presented in Appendix S2.3.

### Demographic pathways of climate suitability effects on λ and time to quasi‐extinction

**Figure 5:**
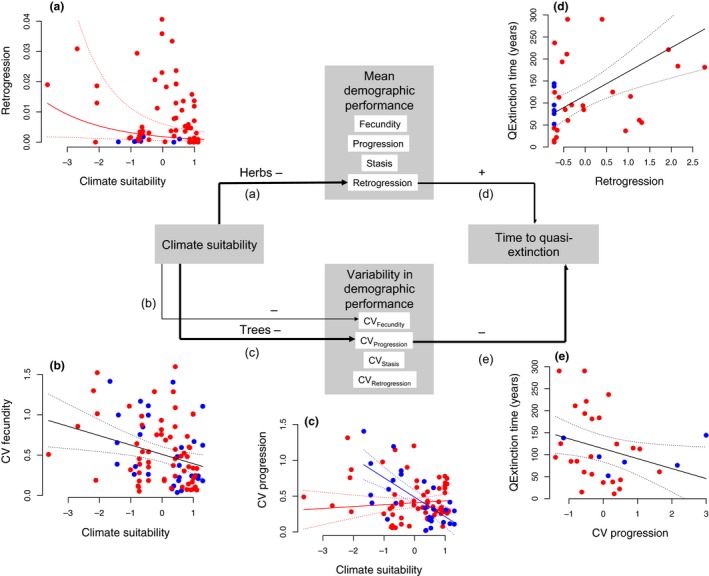
Demographic pathways of the climate suitability effect on population extinction risk (time to 95% probability of quasi‐extinction) via its effects on means and temporal variability of constituent demographic processes. Demographic processes are listed within white boxes, negative and positive signs represent the signs of effects in the best fit models detailed in Table 1 and Appendix S2.3 and represented graphically in panels (a–e). Effect sizes are comparatively presented in Fig. 4. Only variables for which the coefficient confidence intervals did not overlap with zero are shown. CV = coefficient of variation across annual censuses, i.e. temporal variability in demographic performance. Bold arrows represent support for links between climate suitability and extinction resistance via impact of climate on mean and/or variability in demographic performance: mean retrogression increases in relatively unsuitable climates, and the ability to retrogress improves extinction resistance (demographic resistance pathway); temporal variation in progression increases in relatively unsuitable climates, which may negatively impact on extinction resistance (demographic vulnerability pathway). Climate suitability and demographic rate values are centered on zero with unit variance (panels a–e). In panels (d)–(e) the number of populations was limited to *N* = 31 populations for which the projected quasi‐extinction time was < 300 years. Trees are represented with blue, herbaceous perennials with red dots. Dotted lines represent 95% confidence intervals (CI) around the mean. Fitted lines and CI are drawn in blue for trees and in red for herbs when the interaction between growth form and climate suitability was selected in the best model.

Populations in low‐suitability climates had higher mean rates of retrogression (in herbaceous perennials), as well as higher temporal variability in progression (in trees) and fecundity (in both growth form) (Figs 4 and 5a‐c, Table 1, Appendix S2.3). While mean and temporal variation in stasis were selected in the best models and were negatively related to climate suitability, the effect size was very small and confidence intervals encompassed zero (Fig. 4). Mean retrogression was positively, while temporal variation in progression (CV_progression_) was negatively correlated with time to quasi‐extinction (Fig. 5e–d, Appendix S2.3).

Climate suitability was correlated with the elasticity of λ to demographic processes but in interaction with study duration, MPM dimension and λ_*iid*_ (Table 1, Appendix S2.3 and S2.4). Consequently, the importance of demographic rates for population growth rate changed with climate suitability depending on matrix model properties such as study length, as well as factors not necessarily related to climate that affected λ. The elasticity of population growth rate to fecundity was positively correlated with climate suitability when λ_*iid*_ was high and for longer‐lasting studies. The elasticity of λ to retrogression was weakly positively correlated with climate suitability for short studies, but the effect was strongly negative for longer studies. The elasticity of λ to progression was negatively associated with climate suitability for small matrix dimensions and weakly positively related to climate suitability for large matrix dimensions. The elasticity of λ to stasis was positively correlated with climate suitability for small matrices, and weakly negatively correlated with climate suitability for large matrices. The effect sizes for elasticities, and in particular with regard to progression and stasis, were very small, and for the latter two confidence intervals encompassed zero.

The explanatory power of the best models that retained climate suitability was variable (*R* ^2^_fixed effects_ 3–51%; Table 1).

## Discussion

Climate suitability predicted from SDMs fitted with occurrence‐only data and macroclimatic variables affected the mean and temporal variation in demographic processes that underlie population growth and persistence. Less suitable climates constrained population performance, with a lower range of population growth rates and lower range of potential population size responses to perturbations. However, at the spatial and temporal resolution of this study, we found no relationship between climate suitability and mean population performance when expressed as long‐term population growth rate or projected time to quasi‐extinction.

Most populations, across a wide range of climate suitability values, had positive population growth rates and low extinction risk. This supports previous evidence that populations within species’ geographic distribution range are often within the species’ ecological niche estimated with global climate variables (Lee‐Yaw *et al*. 2016). When located within species’ climatic niche envelope, populations can maintain stability across combinations of environmental conditions *via* multiple strategies, including plastic demographic compensation (Villellas *et al*. 2015) and density‐dependent processes (Haldane 1956; Dahlgren *et al*. 2014).

Evidence exists that local extinctions are more frequent under unfavourable conditions (Araújo *et al*. 2002), yet we found little direct support for increasing extinction risk in less suitable climates. These results should spur closer, longer‐term examinations of the mechanisms of extinction in suboptimal climates, which may involve interaction among species’ life‐history strategies, the general macroclimate and local abiotic and biotic conditions (Dullinger *et al*. 2012; Hylander & Ehrlén 2013; Shoemaker *et al*. 2013). Populations with positive growth rates and long times to quasi‐extinction may persist in relatively unsuitable macroclimates due to long extinction lags and/or location in suitable microhabitats which are not modelled by global SDMs (Dullinger *et al*. 2012; Hylander & Ehrlén 2013).

A wider range of population growth rates, both positive and negative, occurred in more suitable climates, where populations also exhibited greater potential for transient boom and bust. The capacity to respond to perturbations may be of particular importance for population establishment (Iles *et al*. 2016) or recovery. Recovery rates after disturbance might be very slow in less suitable climates, potentially leading to long‐term disequilibrium dynamics (Svenning & Sandel 2013) or even extinctions. In more suitable climates populations could capitalize on the benefits of high reproductive rates and fast progression to adulthood to ensure longer‐term persistence. Mirroring this hypothesis, the elasticities of population growth rate to changes in demographic processes varied across the climate suitability gradient. If we consider long studies and high matrix dimension as the most informative in modelling population processes with MPMs, then population growth rates were more elastic to fecundity in highly suitable climates, where large population increases and declines were possible, and more elastic to retrogression in less suitable climates, where increased retrogression enabled long‐term survival of populations.

Further investigations are needed to shed light on the generality of these observations across a larger selection of life histories and additional spatially replicated demographic studies when they become available. If local interactions dominate species distributions in suitable climates (but see Louthan *et al*. 2015), then SDMs fitted with macroclimate and species presences will not be able to predict population growth rate and extinction risk at individual locations (Thuiller *et al*. 2014). Rather than the global macroclimate, it is mid‐ to small geographical scale conditions (i.e. habitat types, biotic interactions, environmental disturbances) that drive significant amounts of variation in asymptotic and transient population dynamics, processes often effective within less than 10 km^2^, the scale of our study (Diez & Pulliam 2007; Coutts *et al*. 2016). However, local‐scale processes might be influenced by the global macroclimate (Louthan *et al*. 2015) and our results suggest that generalizations about demographic responses to mixed signals can be made.

### Demographic pathways of climate suitability effects on population performance

We found increased mean retrogression and increased temporal variability of fecundity and progression in less suitable climates. Increased retrogression has been suggested to be a stress‐tolerance strategy (Salguero‐Gómez & Casper 2010), and in our dataset we found evidence that the ability of plants to retrogress may improve resistance to local extinctions. These results support the hypothesis that progression, i.e. investment in biomass accumulation, rather than fecundity, as well as retrogression, i.e. temporary loss of modules, enhances long‐term persistence of trees and herbaceous perennials and may represent forms of demographic resistance to climatic changes. A wide range of phenotypic responses may improve the chances of local persistence in plant populations affected by climatic constraints (e.g. Valladares *et al*. 2014), retrogression being probably just an extreme solution. In contrast, higher temporal variability in demographic processes in less suitable climatic conditions may signal increased climatically induced stochasticity or lack of adaptation to the environment (Gerst *et al*. 2011). Temporal variation in progression had a detectable negative impact on population extinction resistance and could potentially increase the extinction risk of populations in less suitable climates, suggesting a possible pathway of demographic vulnerability to low‐suitability climates in long‐lived organisms.

Our results are consistent with the idea that demographic processes may trade‐off across different combinations of climate conditions, leading to population stability over large geographic scales (Villellas *et al*. 2015). The use of demographic models of good resolution enabled us to reveal, for a wide range of species, that the effect of climate suitability on population extinction risk is mediated through its effect on the balance of underlying demographic processes. This finding extends the relatively static predictions of SDMs to more dynamic changes in populations brought about by macroclimate change. This has important implications for population persistence, extinction and management in climates that are, or become, unsuitable for species occurrence.

### Limitations and future directions

The biggest challenge in the endeavour of linking predictions of presence‐only SDMs to population performance is finding the climate suitability thresholds beyond which populations start to decline irrecoverably (Doak & Morris 2010). For this, we need to study a larger number of declining populations in less suitable climates; such populations at low densities usually do not make good candidates for long‐term demographic studies, and are underrepresented in global observational datasets, including ours (see also Thuiller *et al*. 2014). Overcoming this major limitation necessitates expanding long‐term population study design to include populations at the edge of species’ ecological niches, designing demographic transplant experiments outside the geographic range limits and planning demographic observational studies over larger temporal and spatial scales, currently among the biggest challenges of spatial demography (Buckley *et al*. 2010; Ehrlén & Morris 2015; Lee‐Yaw *et al*. 2016).

The detection of demographic signal in macroclimate‐based predictions may be hindered by known weaknesses of correlative SDMs. A source of uncertainty may come from not capturing physiologically meaningful climate variables for all species or meaningful interactions between macroclimate variables (Mod *et al*. 2016). In addition, the temporal mismatch between the available climate data and the timing of demographic censuses represents an important source of uncertainty. We fitted our models using the macroclimate data most often employed in SDMs, i.e. WORLDCLIM data averaged over the 1950–2000 period (Hijmans *et al*. 2005). In our demographic dataset, which is a subset of the most detailed demographic observations recorded to date, some studies started as early as 1937 and others ended as late as 2006, while the average study length was just 3.1 ± 1.4 (SD) years. As a result, using climate averages could have potentially hindered our ability to detect important pulses in demographic processes caused by extreme weather effects. An alternative would be to build SDMs by matching climatic variables to the recorded time of the occurrence data (Wisz *et al*. 2015). However, our primary goal here was to test the effects of climate suitability as estimated using SDMs based on climate averages, given the ubiquity of their use for projecting future distributions. Refined methods for species distribution modelling are developed at a rapid rate, providing new frameworks for linking demographic processes to macroclimate suitability in the future.

The notable temporal variability in population performance driven by local factors along with the short time window of most demographic observations will make it challenging to estimate population viability at the scale at which global climate operates (Buckley *et al*. 2010; Crone *et al*. 2013). In our dataset, much temporal variation in demography was likely left unrevealed due to the short study duration relative to the lifespan of the studied organisms. Hierarchical modelling frameworks are a promising avenue to address this challenge in species distribution modelling, in which the scale of interactions between environment and organisms determines the type of data fed into the models (Diez & Pulliam 2007; Evans *et al*. 2016). With this study we promote the capabilities of matrix population modelling in examining predictions of correlative SDMs and more broadly, demography–environment relationships, while we admit that there is much room for further improvement of both SDMs and MPMs in the future.

Finally, any relationship revealed here between climate suitability and demographic performance relies on existing data sources and the quality of the tests is lower than that which could be achieved using a carefully designed demographic monitoring of multiple species across broad climate suitability gradients. Therefore, the observed correlations and related explanatory hypotheses presented here should be further tested using carefully designed experimental and observational approaches. Our theoretical extinction risk estimates in particular deserve validation in the field and further input from minimum viable population analyses (Shoemaker *et al*. 2013).

## Conclusions

Persisting populations observed throughout a wide range of predicted climatic suitability values support the view that species’ broad distribution patterns are, at least in part, linked to climate‐driven variation in population processes. Basic demographic processes may vary across climate suitability gradients, but many populations are able to achieve long‐term persistence through specific demographic mechanisms. We propose that the nature and breadth of these demographic strategies depend on the environmental challenges faced. Macroclimate‐based projections of populations in future climates using SDMs assume that population performance will change as environmental conditions change. Both declining and improving macroclimate conditions can challenge plant species, e.g. increasing climate constraints in populations previously in suitable climates, and increasing pressure from biotic interactions in both suitable and less suitable climates. The demographic strategies achievable by different species and populations will ultimately determine the likelihood of local persistence or extinction. Thousands of correlative SDMs have already been used to make predictions of species’ range shifts with climate change (Chen *et al*. 2011). It is time to examine whether the demographic strategies of persistence available to those species are consistent with these range forecasts. If we do not understand the demographic mechanisms of population persistence under different environmental conditions, we run the risk of overestimating species’ extinction rates in less suitable climates and the persistence probability in more suitable climates.

## Authorship

AMC, RS‐G and YMB developed the initial concept with input from ALA. AMC wrote the first draft of the manuscript, RS‐G developed code and provided support for deriving demographic metrics, RS‐G, SRC and YB advised on demographic modelling, OB provided code and technical support for species distribution modelling, AG and OB advised on species distribution modelling, YMB and SRC advised on statistical analysis. EW provided digitized Meusel‐Jäger atlas maps and BJE, BM, JCS, CV and the BIEN working group provided BIEN occurrence data. All authors made substantial contributions to editing the manuscript and interpretation of results.

## Data Accessibility Statement

Data available from the Dryad Digital Repository: https://doi.org/10.5061/dryad.2js00.
