# Supplementary material for: Less favourable climates constrain demographic strategies in plants
Source: Ecol Lett. 2017 Jun 13;20(8):969–80. doi: 10.1111/ele.12794 (PMC5575490; doi:10.1111/ele.12794)
Supplement: Supplementary file 1 [file ELE-20-969-s001.pdf]

Supporting Information Appendix S1

Appendix S1.1. Data selection

Data quality was a primary concern and significant effort was spent to ensure that demographic, species occurrence and climate data were at appropriate scales and as accurate as possible. The demographic data selection resulted in a first set of 123 species with 311 populations in COMPADRE 3.0.0 (Salguero-Gómez et al. 2015). Of these, 34 species across 93 populations had presence data adequate for fitting quality Species Distribution Models, which resulted in our final dataset (Figure S1.1 and Table S1.1).

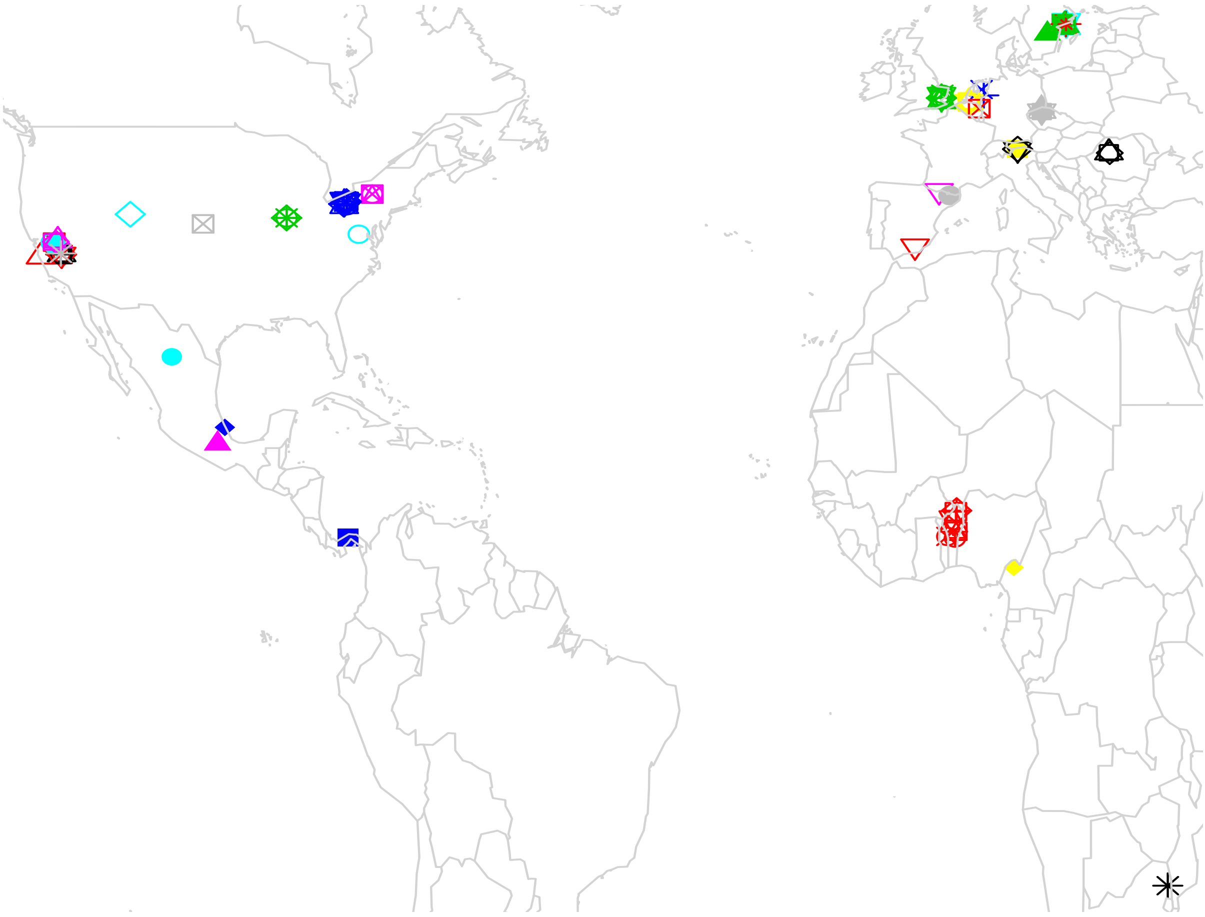

Figure S1.1. Location of the 34 species with 93 populations selected for this study. Combinations of different symbols and colours indicate different species.

Demographic data selection. Population matrix models were extracted from the COMPADRE Plant Matrix Database version 3.0.0 accessed in July 2014 from www.compadre-db.org (Salguero-Gómez et al. 2015). We imposed the following species and study selection criteria: (1) field observations were carried out for at least three years (two matrix transitions), 2) matrices were under control conditions (experimental manipulations were excluded) (3) individual matrices were reported for each year and population separately so that we were able to compute stochastic demographic metrics and measures of temporal variability, (4) the study was conducted within the species' native geographic range (5) the geographic location of populations was reported with a coordinate accuracy of at least 10 km (i.e., at least one digit) to ensure matching with grid size of environmental variables, (6) matrices were complete, with all vital rates, including fecundity, recorded, (7) matrices were quality checked i.e., survival was <1.09 in "survival issue" column in COMPADRE (stage-specific survival probabilities cannot be much greater than 1, see Salguero-Gómez et al. 2015) and matrices were not suspected of seed stage error i.e., fecundity was reported as the fate of organisms over a time interval and not simple seed count, (8) matrices were irreducible and ergodic (see Stott et al. 2010 for details) (9) species belonged to either the "tree" or "herbaceous perennial" growth types (other growth forms were excluded due to low sample size after all other selection criteria). Precision of population coordinates was crosschecked and verified on Google Earth for all populations where it was possible based on the original paper. Subsequently another seven species (eight populations) with study length longer than 25 years and matrix dimension higher than

14 were eliminated because they were strong outliers relative to the study length and matrix dimension of other populations.

Species occurrence data selection. Overall, the incomplete and sometimes poor quality data of global biodiversity repositories limited the number of species selected from COMPADRE for which we could fit quality distribution models. To overcome the reliability issue of single-source occurrence data for producing realistic distribution maps (Duputié et al. 2014), we compiled the dataset from two major global and continental data repositories: GBIF (Global Biodiversity Information Facility, accessed on 28 November 2014) and BIEN (Botanical Information and Ecology Network, www.bien3.org[1], accessed on 16 November 2014), from local and regional herbaria and digitized species distribution maps from atlases (Meusel and Jäger 1992) (Table S1.1 below). There are a number of limitations to using occurrence records from big data repositories in species distribution modeling (Newbold 2010), therefore we carefully cleaned the GBIF, BIEN and regional herbaria data and selected locality records as reliably as possible, including only occurrences reported to at least one digit (~10 km) accuracy, with a coordinate precision of 10 km or no reported precision, and deleting cultivated specimens, non-native occurrences and duplicated records. For species outside Eurasia, we approximated based on independent descriptions whether the occurrence dataset covered roughly the whole species area of distribution, and if gaps spanning > *ca.* 75% of the range were identified, we assumed that the probability of the existing dataset representing the species' ecological requirements was low and the

[1] http://bien.nceas.ucsb.edu/bien/; http://vegpath.org/BIEN3/wiki/conditions_of_use;

species was omitted. In Eurasian taxa, where global datasets were incomplete we used digitized species distribution maps from atlases to ensure we covered species' full geographic range (Meusel and Jäger 1992). We extracted all presence points reported as such and another set of presence points from a large number of random points within the polygon of the distributional range (see maps below for a visual comparison of occurrence data completeness of the pooled GBIF and BIEN datasets and the Meusel-Jäger dataset for species with available maps in our dataset).

To reduce spatial autocorrelation due to sampling bias, occurrences closer to each other than 10 km were removed using a disaggregation index in the *ecospat* R package (Broennimann et al. 2015). Various taxa were checked for taxonomic consistency across the different data sources and infrataxa with poorly known geographic distribution were not modeled (e.g., *Anthyllis vulneraria* ssp. *alpicola*).

Taxa for which the final number of occurrences was lower than 30 and/or were suspected of not covering the range of environmental conditions across the area of distribution were not used because of increased risk of biased predictions (Wisz et al. 2008). The minimum number of occurrence points available per species was 56 (Table S1.1), ensuring sufficient modeling power. We did not use the COMPADRE population locations to build SDMs and we deleted any occurrence points that overlapped with COMPADRE populations.

Table S1.1. Summary of data sources used in Species Distribution Modeling for 34 species of trees and herbaceous perennials. Numbers represent cleaned occurrences subsequently subject to disaggregation (see text above for details on data cleaning). GBIF = Global Biodiversity Information Facility (http://www.gbif.org), BIEN = Botanical Information and Ecology Network

(http://bien.nceas.ucsb.edu/bien/), Meusel = digitised distribution maps (Meusel and Jäger 1992; http://www2.biologie.uni-halle.de/bot/ag_chorologie/choro/index.php?Lang=E), Meusel_dots = occurrence data represented as distinct localities on the Meusel maps, Meusel_poly = points randomly selected from within the polygon representing the distribution area of a species on the Meusel maps (Meusel_dots excluded). The number of points was selected to complement the cleaned and disaggregated occurrence dataset (GBIF, BIEN and the other sources) up to 10,000 occurrences. NHM = Hungarian National History Museum (http://www.nhmus.hu/en/english_home), Researcher = researcher's own digitized data. GF=Growth form, T=Tree, HP=Herbaceous perennial. Final input data are available to download from Dryad. The full reference for each species is provided separately at the end of this document.

Species	Reference	GBIF	BIEN	Meusel.dots	Meusel.poly	NHM	Researcher	GF
Abies concolor	Van Mantgem and Stephenson 2005	223	53846	-	-	-	-	T
Abies magnifica	Van Mantgem and Stephenson 2005	41	15163	-	-	-	-	T
Acer saccharum	Lin and Augspurger 2008	469	610189	-	-	-	-	T
Actaea spicata	Fröborg and Eriksson 2003	13985	4	5015	3133	69	-	HP
Alnus incana rugosa	Huenneke and Marks 1987	391	233	-	-	-	-	T
Artemisia genipi	Marcante et al. 2009	64	0	-	-	-	-	HP
Aster amellus	Munzbergová 2007	661	0	313	9541	5	-	HP
Calocedrus decurrens	Van Mantgem and Stephenson 2005	177	8804	-	-	-	-	T
Calochortus albus	Fiedler 1987	50	32	-	-	-	-	HP
Cirsium dissectum	Jongejans et al. 2008	3406	0	12359	-	-	-	HP
Cryptantha flava	Lucas et al. 2008	132	154	-	-	-	-	HP
Cypripedium calceolus	García et al. 2010	1020	8	13443	5904	-	-	HP
Echinacea angustifolia	Hurlburt 1999	600	302	-	-	-	-	HP
Heteropogon contortus	O'Connor 1993	2787	14	-	-	-	-	HP
Khaya senegalensis	Gaoue and Ticktin 2010	56	0	-	-	-	-	T
Lathyrus vernus	Ehrlén 1995	9942	0	72	8617	11	-	HP
Manilkara zapota	Cruz-Rodríguez et al. 2009	631	473	-	-	-	-	T
Mimulus cardinalis	Angert 2006	167	55	-	-	-	-	HP
Mimulus lewisii	Angert 2006	377	228	-	-	-	-	HP
Molinia caerulea	Jacquemyn et al. 2005	77404	0	47	5265	18	-	HP
Pinus lambertiana	Van Mantgem and Stephenson 2005	79	3899	-	-	-	-	T
Poa alpina	Marcante et al. 2009	6525	452	7404	1308	-	-	GP
Primula veris	Endels et al. 2005; Ehrlén et al. 2005	38791	0	98	7915	26	-	HP
Primula vulgaris	Valverde and Silvertown 1998	5021	4	166	9038	54	-	HP
Prioria copaifera	Condit et al. 1993	103	136	-	-	-	-	T
Prosopis glandulosa	Golubov et al. 1999	229	1698	-	-	-	-	T
Prosopis laevigata	Bernal 2004	226	42	-	-	-	-	T
Prunus africana	Steward 2001	146	0	-	-	-	-	T
Ramonda myconi	Picó and Riba 2002	369	0	-	-	-	-	HP
Saponaria bellidifolia	Csergő et al. 2011	82	0	-	-	-	71	HP

Sarcocapnos enneaphylla	Salinas et al. 2002	475	0	1342	-	-	-	HP
Succisa pratensis	Mildén 2005	48984	0	23	7366	1	-	HP
Trillium grandiflorum	Knight 2003	261	386	-	-	-	-	HP
Tsuga canadensis	Lamar and McGraw 2005	371	194269	-	-	-	-	T

Appendix S1.2. Calculation of population growth rates, time to quasi-extinction, transient population dynamics and demographic processes (matrix transitions)

Mean and variation of population growth rate (λ). Matrix projection models (MPMs) were used to calculate five different measures of population growth rate. Two mean deterministic measures were calculated for each population: the arithmetic mean of individual population growth rate estimates over time (λ_{arith}) and the geometric mean of individual population growth rate estimates over time (λ_{geom}). In contrast to deterministic population performance metrics stochastic metrics include the effect of observed temporal variability in estimates. We calculated stochastic population growth rate (λ_{iid}) for each population by simulation. We started from the stable stage distribution i.e., a theoretical state in which the proportion of individuals in each developmental stage remains constant over time and projected population growth over 50,000 time intervals by randomly choosing one of the individual matrix models recorded for a population. λ_{iid} was the arithmetic mean of $log[N_{(t+1)}/N_{(t)}]$ of each successive interval over all time steps (Morris and Doak 2002). To account for differences in the effect of the temporal variation in individual matrix entries on population growth, we calculated Tuljapurkar's analytic approximation for population growth rate, λ_{Tulja}, which estimates the stochastic population growth rate from matrices and covariances among matrix entries

(Tuljapurkar 1982). However because this method assumes that the variation among individual matrices is low, results need cautious interpretation (Morris and Doak 2002). Both the simulation and Tuljapurkar's analytic approximation assume lack of temporal autocorrelation between matrix elements and population growth rates (i.e., identical and independently distributed environments). We estimated a third, sequential growth rate (λ_{seq}) in temporally autocorrelated environments. For this metric, each species' population, the population matrix models were used in the same sequence that they were used from data from the field, and λ_{seq} was calculated by taking the *n*-th root of the dominant eigenvalue of the matrix product [$\mathbf{A}_n\mathbf{A}_{n-1}\ldots\mathbf{A}_1$], where **A** represent individual matrices over time and *n* the total number of matrices per population (Buckley et al. 2010). All population growth rate estimates were highly correlated (Spearman's rho=0.96-0.99).

Time to quasi-extinction. We estimated the probability of local extinction by simulating population densities over time with stochastic quasi-extinction probability curves (Morris and Doak 2002 Box 7.5). Extinction probability estimations require *a priori* knowledge of total population size and individual numbers in each stage (i.e. initial population vector $\boldsymbol{n_0}$, Morris and Doak 2002). Because $\boldsymbol{n_0}$ values are not regularly reported and are not archived in COMPADRE (Salguero-Gómez et al. 2015), we initially chose a range of hypothetical initial population sizes between 1- 200 aboveground individuals, multiplying each by the stable stage distribution to obtain extinction estimates from the asymptotic demographic stage structure in a particular habitat. We then determined the quasi-extinction probability of each population and stopped the simulations at 300 years. The

extinction risk averaged over all 200 hypothetical population sizes was highly correlated with values calculated for 200 individuals (Spearman's rho = 0.850) and consequently we report results only for the latter. To obtain robust estimates, extinction probability curves were averaged over 10 separate simulations, with each simulation based on 5,000 iterations.

Transient population dynamics. Disturbances like fire, extreme weather, disease epidemics, pest outbreaks or human activity can change a population's age-, size- or stage structure if some stages are affected more than others. When a population is not at stable state, it can exhibit rates of growth that are faster (population 'amplification'), or slower (population 'attenuation') than predicted under stable conditions. These phenomena are due to a relative over- or under-representation of reproductive individuals compared to the stable structure. Transient bounds (Stott et al. 2011) describe the limits of these dynamics: the largest or smallest population size achievable following a disturbance, relative to size of the population in the absence of disturbance (i.e. growing at stable asymptotic rate). We calculated four bounds: the upper and lower bounds on reactivity, which respectively describe the largest and smallest possible abundance relative to stable state in the first time step of projection immediately following a disturbance and the upper and lower bounds on inertia, which respectively describe the largest and smallest ratios of population abundance relative to stable state that a population may settle to *ad infinitum* as a result of disturbance. We determined the range of transient dynamics which can occur in the first time step and *ad infinitum*

respectively using *log(upper bound)* - *log(lower bound)* for each of reactivity and inertia. The indices were calculated from the element-by-element arithmetic mean MPM for each population.

Demographic processes (matrix transitions). Matrix population models compiled in COMPADRE are constructed from field measurements and represent the proportion of individuals that transition across different size-, stage or age categories in a standard format across species (Salguero-Gómez et al. 2015). From each projection matrix we extracted the following basic demographic processes: fecundity, progression, retrogression and stasis (Silvertown et al. 1993). Fecundity represents reproductive effort and/or recruitment given that plants have survived, progression is when plants survive and grow, retrogression is when plants survive but lose some modules or shrink in size, stasis is when plants survive but neither grow or shrink. Clonality was not reported separately for either species. We calculated a weighted average of the corresponding matrix entries in the transition matrix. Weights corresponded to relative abundances of each stage at stationary equilibrium, retrieved from the right eigenvector of the projection matrix (the stable stage distribution). For example, to calculate mean progression in Figure S1.2, we calculated the sum of the entries in the corresponding columns in green, which were multiplied by the corresponding element of the stable stage distribution.

	s	v1	v2	r
s	0.05	0	0	0.119
v1	0.35	0.846	0.091	0.017
v2	0	0.077	0.364	0.051
r	0	0	0.545	0.932

Progression
Stasis
Retrogression
Fecundity

Figure S1.2. Illustration of demographic processes (matrix transitions) calculated from specific entries in the Matrix Projection Model. This sample transition matrix is built from categories of developmental stages (s=seedlings, v1=small vegetatives, v2=large vegetatives, r=reproductives) and numbers represent the proportion of individuals experiencing transitions between categories. Different colours represent matrix regions corresponding to progression, stasis, retrogression and fecundity respectively.

Appendix S1.3. Species Distribution Modeling approach

Study extent

In comparative distribution modeling studies, it is important to choose a large enough area, covering the niche of the modeled species that has been accessible to the species over relevant time periods (Barve et al. 2011). We first delimited the study extent broadly to native biomes. Then, we performed a Multivariate Environmental Similarity Surface (MESS) analysis to identify and select only biome grid cells that have climates analogous to the ecoregions occupied by the species (Elith et al. 2010). Ecoregions are smaller biogeographical units than biomes, have more homogeneous climates and similar vegetation types and lack major geographical barriers to species dispersal. We thus avoided extrapolating SDM predictions to non-analog climates, which may provide

spurious and unreliable results due to changes in multicollinearity of predictors (Fitzpatrick and Hargrove 2009, Peterson 2011, Guisan et al. 2014).

Climatic variable selection. We selected a limited set of eight environmental predictors for all species, commonly used in distribution models for plant species (e.g., Thuiller et al., 2005, Broennimann et al., 2007, Petitpierre et al., 2012, Mod et al. 2016): annual mean temperature, temperature seasonality, mean temperature of warmest quarter, mean temperature of coldest quarter, precipitation seasonality and precipitation of wettest quarter, extracted from the WORLDCLIM – Global Climate Data portal (http://www.worldclim.org/bioclim; Hijmans et al. 2015) and annual and seasonality (standard deviation of monthly values) of global potential evapo-transpiration, extracted from the CGIAR Consortium for Spatial Information (http://www.cgiar-csi.org/data). We downloaded the maps at 5 arc-minutes resolution (~10 km in the temperate regions), assuming the scale is suitable to detect macroclimate drivers of demographic processes. With this scale, we tolerated uncertainties in reported geographic coordinates in COMPADRE (Salguero-Gómez et al. 2015).

Modeling approach

As species distribution models are sensitive to different analytical approaches (Elith et al. 2006), we used an ensemble of four different techniques: generalized linear models (GLM), generalized boosted regression models (GBM; Friedman et al., 2000), random forest (RF, Breiman 2001) and maximum entropy modeling (MAXENT; Phillips et al. 2006) with no interaction between terms and all other parameters set to their default

values as implemented in the BIOMOD2 library (Thuiller et al. 2009). A large number of pseudo-absences were randomly sampled from the study area for each species (see Table S1.1. for details) and a weight was applied in the calibration of the models so that presences and pseudo-absences have equal (0.5) prevalence. Models were calibrated with 70% of the data and evaluated with the remaining 30% of the data. The procedure was replicated 25 times per species with random training and evaluation datasets, adding up to 100 models per species (4 techniques x 25 runs each). We compared four different evaluation metrics: Roc AUC (Fielding and Bell 1997), TSS (Allouche et al. 2006), KAPPA (Allouche et al. 2006) and the "presence-only" evaluator Boyce index (Boyce et al. 2002, Hirzel et al. 2006) to assess model performance. The final ensemble model was constructed from selected models with the evaluation metric TSS larger than 0.7, by taking a weighted average proportional to the TSS values. A coefficient of variation was calculated across runs and techniques for the final model and across runs for each technique and the spatial distribution of model uncertainty was visually inspected. We produced a separate ensemble model for each technique for visual comparison, by weighted averaging across all runs.

The species distribution models (SDMs) of the final species set had generally high predictive power with high values of the evaluation metrics (TSS = 0.860±0.060; 0.851; AUC = 0.978±0.016; 0.98; KAPPA =0.673±0.176; 0.749; Boyce = 0.984±0.017; 0.987) (mean ± 1SD; median). There was no major difference between the prediction accuracy of models built with and without data from Meusel maps (TSS_{Meusel} = 0.845 ± 0.044; n=11 species and $TSS_{non\text{-}Meusel}$ = 0.866 ± 0.067; n=23 species) (mean ± 1SD). For a few

species (e.g. *Potentilla anserina, Oxalis acetosella, Eremophila forrestii forrestii, Cirsium undulatum*) none of the model runs reached the selected TSS threshold of 0.7, therefore these species were not considered in further analyses.

Occurrence data for each species and SDM predictions along with the position of COMPADRE populations on the projected climate suitability maps are presented in Supporting material uploaded to Dryad.

References (Table S1.1 of this document)

1.

Angert, A.L. (2006). Demography of central and marginal populations of monkeyflowers (Mimulus cardinalis and M-lewisii). *Ecology*, 87, 2014-2025.

2.

Bernal, R. (2004). Demografia de Prosopis laevigata, principal hospedero de Tillandsia recurvata. PhD Thesis.

3.

Condit, R., Hubbell, S.P. & Foster, R.B. (1993). Mortality and Growth of a Commercial Hardwood El-Cativo, Prioria-Copaifera, in Panama. *Forest Ecol Manag*, 62, 107-122.

4.

Cruz-Rodriguez, J.A., Lopez-Mata, L. & Valverde, T. (2009). A comparison of traditional elasticity and variance-standardized perturbation analyses: a case study with the

tropical tree species Manilkara zapota (Sapotaceae). *J Trop Ecol*, 25, 135-146.

5.

Csergo, A.M., Molnar, E. & Garcia, M.B. (2011). Dynamics of isolated Saponaria bellidifolia Sm. populations at northern range periphery. *Popul Ecol*, 53, 393-403.

6.

Ehrlen, J. (1995). Demography of the Perennial Herb Lathyrus-Vernus .2. Herbivory and Population-Dynamics. *Journal of Ecology*, 83, 297-308.

7.

Ehrlen, J., Syrjanen, K., Leimu, R., Garcia, M.B. & Lehtila, K. (2005). Land use and population growth of Primula veris: an experimental demographic approach. *Journal of Applied Ecology*, 42, 317-326.

8.

Endels, P., Jacquemyn, H., Brys, R. & Hermy, M. (2005). Rapid response to habitat restoration by the perennial Primula veris as revealed by demographic monitoring. *Plant Ecol*, 176, 143-156.

9.

Fiedler, P.L. (1987). Life-History and Population-Dynamics of Rare and Common Mariposa Lilies (Calochortus Pursh, Liliaceae). *Journal of Ecology*, 75, 977-995.

10.

Froborg, H. & Eriksson, O. (2003). Predispersal seed predation and population dynamics in the perennial understorey herb Actaea spicata. *Can J Bot*, 81, 1058-1069.

11.

Gaoue, O.G. & Ticktin, T. (2010). Effects of Harvest of Nontimber Forest Products and Ecological Differences between Sites on the Demography of African Mahogany. *Conservation Biology*, 24, 605-614.

12.

Garcia, M.B., Goni, D. & Guzman, D. (2010). Living at the Edge: Local versus Positional Factors in the Long-Term Population Dynamics of an Endangered Orchid. *Conservation Biology*, 24, 1219-1229.

13.

Golubov, J., Mandujano, M.D., Franco, M., Montana, C., Eguiarte, L.E. & Lopez-Portillo, J. (1999). Demography of the invasive woody perennial Prosopis glandulosa (honey mesquite). *Journal of Ecology*, 87, 955-962.

14.

Huenneke, L.F. & Marks, P.L. (1987). Stem Dynamics of the Shrub Alnus-Incana Ssp Rugosa - Transition Matrix Models. *Ecology*, 68, 1234-1242.

15.

Hurlburt, D.P. (1999). Population ecology and economic botany of Echinacea angustifolia, a native prairie medicinal plant. Department of Ecology and Evolutionary Biology, University of Kansas.

16.

Jacquemyn, H., Brys, R. & Neubert, M.G. (2005). Fire increases invasive spread of Molinia caerulea mainly through changes in demographic parameters. *Ecol Appl*, 15, 2097-2108.

17.

Jongejans, E., de Vere, N. & de Kroon, H. (2008). Demographic vulnerability of the clonal and endangered meadow thistle. *Plant Ecol*, 198, 225-240.

18.

Knight, T.M. (2003). Effects of herbivory and its timing across populations of Trillium grandiflorum (Liliaceae). *American Journal of Botany*, 90, 1207-1214.

19.

Lamar, W.R. & McGraw, J.B. (2005). Evaluating the use of remotely sensed data in matrix population modeling for eastern hemlock (Tsuga canadensis L.). *Forest Ecol Manag*, 212, 50-64.

20.

Lin, Y. & Augspurger, C.K. (2008). Impact of spatial heterogeneity of neighborhoods on long-term population dynamics of sugar maple (Acer saccharum). *Forest Ecol Manag*, 255, 3589-3596.

21.

Lucas, R.W., Forseth, I.N. & Casper, B.B. (2008). Using rainout shelters to evaluate climate change effects on the demography of Cryptantha flava. *Journal of Ecology*, 96, 514-522.

22.

Marcante, S., Winkler, E. & Erschbamer, B. (2009). Population dynamics along a primary succession gradient: do alpine species fit into demographic succession theory? *Ann Bot-London*, 103, 1129-1143.

23.

Milden, M. (2005). Local and regional dynamics of Succisa pratensis. PhD Thesis, Department of Botany, Stockholm University.

24.

Munzbergova, Z. (2007). Population dynamics of diploid and hexaploid Populations of a perennial herb Population dynamics of diploid and hexaploid populations of a perennial herb. *Ann Bot-London*, 100, 1259-1270.

25.

Oconnor, T.G. (1993). The Influence of Rainfall and Grazing on the Demography of Some African Savanna Grasses - a Matrix Modeling Approach. *Journal of Applied Ecology*, 30, 119-132.

26.

Pico, F.X. & Riba, M. (2002). Regional-scale demography of Ramonda myconi: Remnant population dynamics in a preglacial relict species. *Plant Ecol*, 161, 1-13.

27.

Salinas, M.J., Suarez, V. & Blanca, G. (2002). Demographic structure of three species of Sarcocapnos (Fumariaceae) as a basis for their conservation. *Can J Bot*, 80, 360-369.

28.

Stewart, K.M. (2001). The commercial bark harvest of the African cherry (Prunus africana) on Mount Oku, Cameroon: Effects on traditional uses and population dynamics. Florida International University ProQuest ETD Collection for FIU.

29.

Valverde, T. & Silvertown, J. (1998). Variation in the demography of a woodland understorey

herb (Primula vulgaris) along the forest regeneration cycle: projection matrix analysis. *Journal of Ecology*, 86, 545-562.

30.

Van Mantgem, P.J. & Stephenson, N.L. (2005). The accuracy of matrix population model projections for coniferous trees in the Sierra Nevada, California. *Journal of Ecology*, 93, 737-747.

References (General)

1.

Allouche, O., Tsoar, A. & Kadmon, R. (2006). Assessing the accuracy of species distribution models: prevalence, kappa and the true skill statistic (TSS). *Journal of Applied Ecology*, 43, 1223-1232.

2.

Barve, N., Barve, V., Jimenez-Valverde, A., Lira-Noriega, A., Maher, S.P., Peterson, A.T. *et al.* (2011). The crucial role of the accessible area in ecological niche modeling and species distribution modeling. *Ecological Modelling*, 222, 1810-1819.

3.

Boyce, M.S., Vernier, P.R., Nielsen, S.E. & Schmiegelow, F.K.A. (2002). Evaluating resource selection functions. *Ecological Modelling*, 157, 281-300.

4.

Breiman, L. (2001). Random forests. *Mach Learn*, 45, 5-32.

5.

Broennimann, O., Treier, U.A., Muller-Scharer, H., Thuiller, W., Peterson, A.T. & Guisan, A. (2007). Evidence of climatic niche shift during biological invasion. *Ecology Letters*, 10, 701-709.

6.

Broennimann, O.P., B.; Randin, C.; Engler, R.; Di Cola, V.; Breiner, F.; D`Amen, M.; Pellissier, L.; Pottier, J.; Pio, D.; Garcia, M. R.; Hordijk, W.; Dubuis, A.; Scherrer, D.; Salamin, N.; Guisan, A.; (2015). ecospat: Spatial Ecology Miscellaneous Methods. R package version 1.1. http://CRAN.R-project.org/package=ecospat.

7.

Buckley, Y.M., Ramula, S., Blomberg, S.P., Burns, J.H., Crone, E.E., Ehrlen, J. *et al.* (2010). Causes and consequences of variation in plant population growth rate: a synthesis of matrix population models in a phylogenetic context. *Ecology Letters*, 13, 1182-1197.

8.

Duputie, A., Zimmermann, N.E. & Chuine, I. (2014). Where are the wild things? Why we need better data on species distribution. *Global Ecol Biogeogr*, 23, 457-467.

9.

Elith, J., Graham, C.H., Anderson, R.P., Dudik, M., Ferrier, S., Guisan, A. *et al.* (2006). Novel methods improve prediction of species' distributions from occurrence data. *Ecography*, 29, 129-151.

10.

Elith, J., Kearney, M. & Phillips, S. (2010). The art of modelling range-shifting species. *Methods in Ecology and Evolution*, 1, 330-342.

11.

Fielding, A.H. & Bell, J.F. (1997). A review of methods for the assessment of prediction errors in conservation presence/absence models. *Environ Conserv*, 24, 38-49.

12.

Fitzpatrick, M.C. & Hargrove, W.W. (2009). The projection of species distribution models and the problem of non-analog climate. *Biodivers Conserv*, 18, 2255-2261.

13.

Friedman, J., Hastie, T. & Tibshirani, R. (2000). Additive logistic regression: A statistical view of boosting. *Ann Stat*, 28, 337-374.

14.

Guisan, A., Petitpierre, B., Broennimann, O., Daehler, C. & Kueffer, C. (2014). Unifying niche shift studies: insights from biological invasions. *Trends in Ecology & Evolution*, 29, 260-269.

15.

Hijmans, R.J., Cameron, S.E., Parra, J.L., Jones, P.G. & Jarvis, A. (2005). Very high resolution interpolated climate surfaces for global land areas. *International Journal of Climatology*, 25, 1965-1978.

16.

Hirzel, A.H., Le Lay, G., Helfer, V., Randin, C. & Guisan, A. (2006). Evaluating the ability of habitat suitability models to predict species presences. *Ecological Modelling*, 199, 142-152.

17.

Meusel, H., Jäger, E. (1992). *Vergleichende Chorologie der Zentraleuropäischen Flora, I-III.* Fischer, Jena.

18.

Mod, H.K., Scherrer, D., Luoto, M. and Guisan, A. (2016). What we use is not what we know: environmental predictors in plant distribution models. *J Veg Sci.*

19.

Morris, W.F.D., Doak, F. (2002). *Quantitative conservation biology: theory and practice of population viability analysis*. Sinauer Associates, Sunderland, Massachusetts, USA.

20.

Newbold, T. (2010). Applications and limitations of museum data for conservation and ecology, with particular attention to species distribution models. *Prog Phys Geog*, 34, 3-22.

21.

Peterson, A.T. (2011). Ecological niche conservatism: a time-structured review of evidence. *Journal of Biogeography*, 38, 817-827.

22.

Petitpierre, B., Kueffer, C., Broennimann, O., Randin, C., Daehler, C. & Guisan, A. (2012). Climatic Niche Shifts Are Rare Among Terrestrial Plant Invaders. *Science*, 335, 1344-1348.

23.

Phillips, S.J., Anderson, R.P. & Schapire, R.E. (2006). Maximum entropy modeling of species geographic distributions. *Ecological Modelling*, 190, 231-259.

24.

Salguero-Gomez, R., Jones, O.R., Archer, C.R., Buckley, Y.M., Che-Castaldo, J., Caswell, H. *et al.* (2015). The COMPADRE Plant Matrix Database: an open online repository for plant demography. *Journal of Ecology*, 103, 202-218.

25.

Silvertown, J., Franco, M., Pisanty, I. & Mendoza, A. (1993). Comparative plant demography–relative importance of life-cycle components to the finite rate of increase in woody and herbaceous perennials. *J. Ecol.*, **81**, 465–476.

26.

Stott, I., Townley, S., Carslake, D. & Hodgson, D.J. (2010). On reducibility and ergodicity of population projection matrix models. *Methods in Ecology and Evolution*, 1, 242-252.

27.

Stott, I., Townley, S. & Hodgson, D.J. (2011). A framework for studying transient dynamics of population projection matrix models. *Ecol Lett*, 14, 959-970.

28.

Thuiller, W., Lafourcade, B., Engler, R. & Araujo, M.B. (2009). BIOMOD - a platform for ensemble forecasting of species distributions. *Ecography*, 32, 369-373.

29.

Thuiller, W., Richardson, D.M., Pysek, P., Midgley, G.F., Hughes, G.O. & Rouget, M. (2005). Niche-based modelling as a tool for predicting the risk of alien plant invasions at a global scale. *Global Change Biol*, 11, 2234-2250.

30.

Tuljapurkar, S.D. (1982). Population-Dynamics in Variable Environments .2. Correlated

Environments,Sensitivity Analysis and Dynamics. *Theor Popul Biol*, 21, 114-140.

31.

Wisz, M.S., Hijmans, R.J., Li, J., Peterson, A.T., Graham, C.H., Guisan, A. *et al.* (2008). Effects of sample size on the performance of species distribution models. *Divers Distrib*, 14, 763-773.

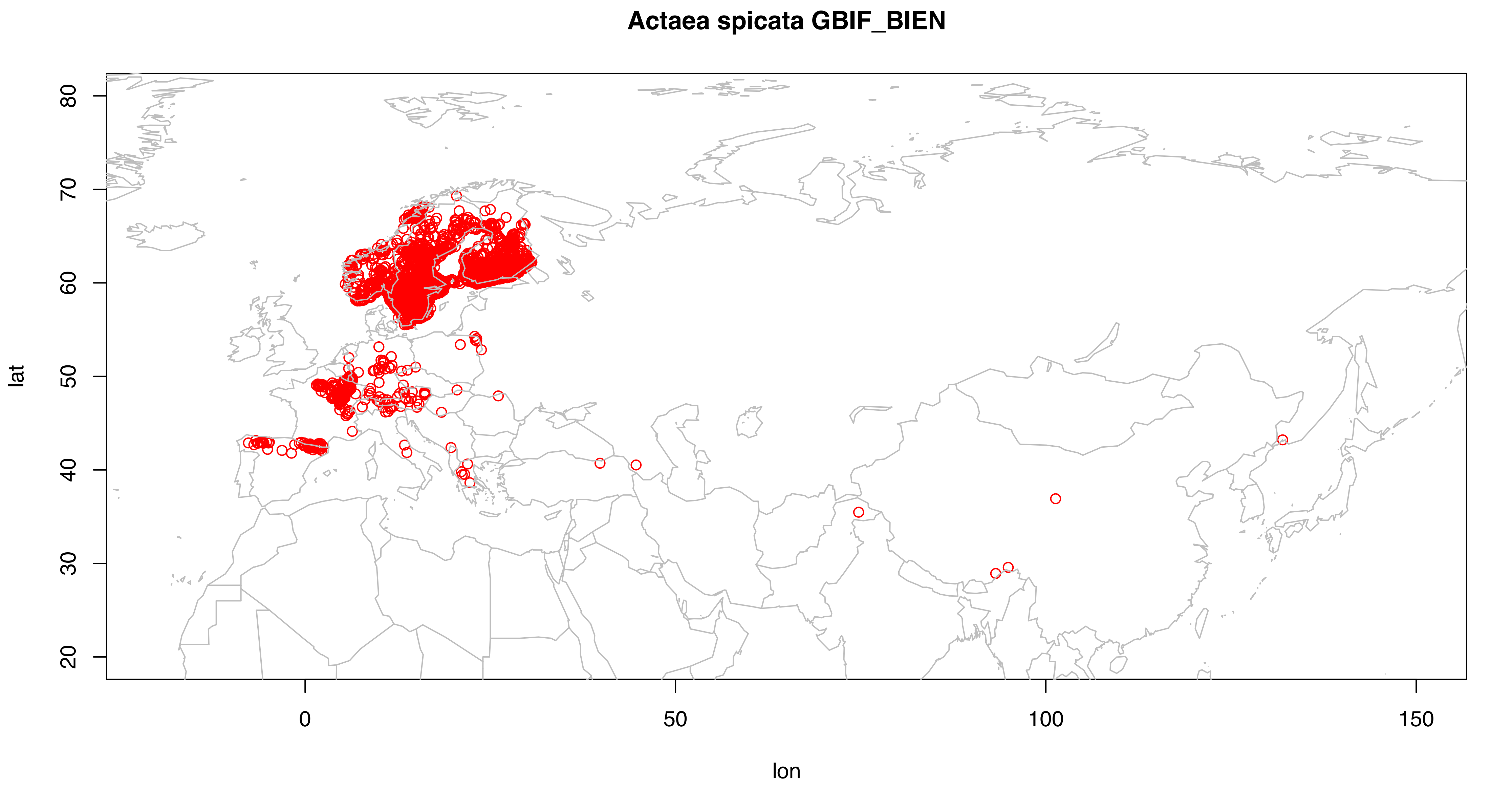

Actaea spicata GBIF_BIEN
lat
lon
80
70
60
50
40
30
20
0
50
100
150

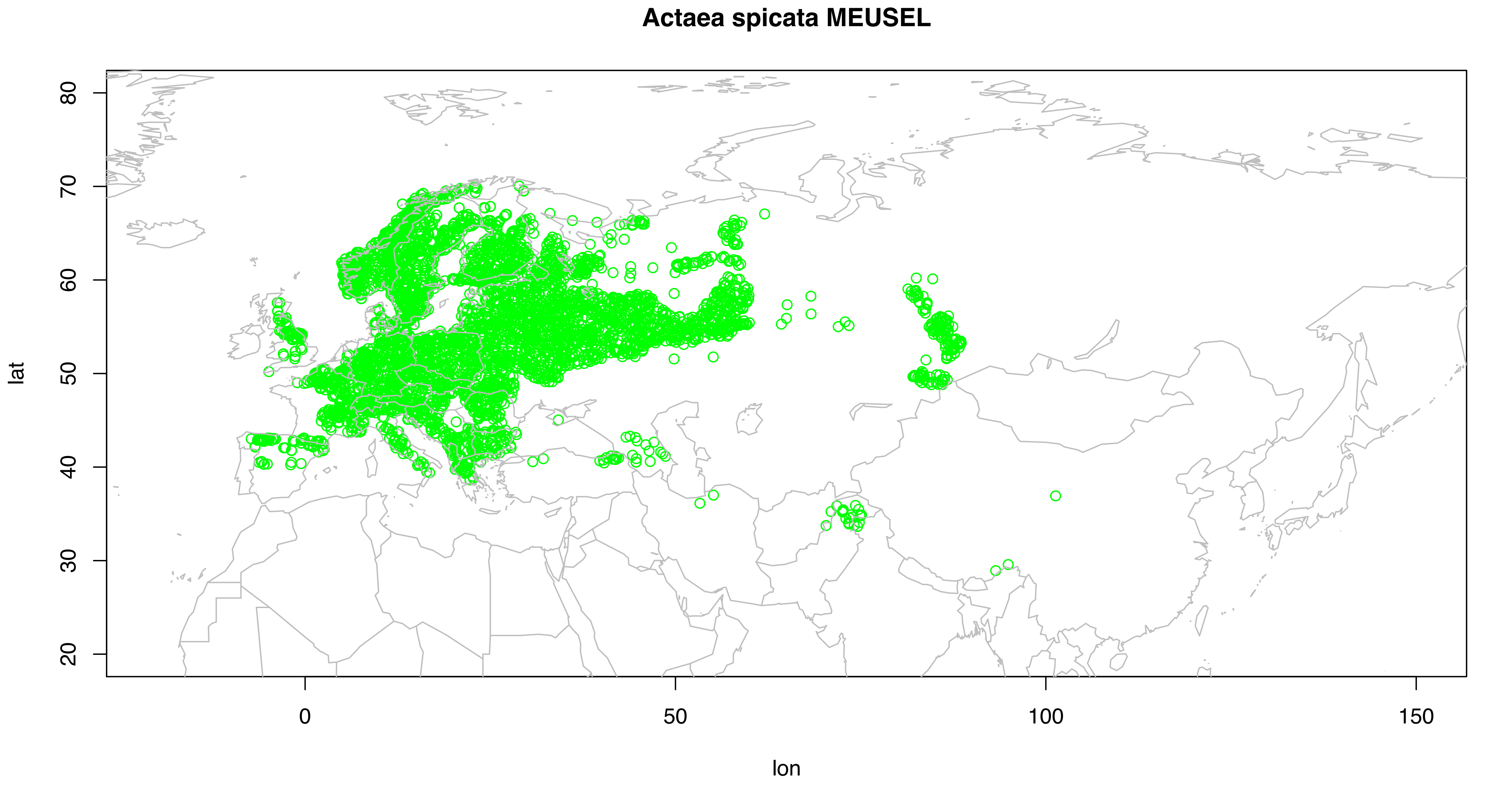

Actaea spicata MEUSEL
lat
lon
80
70
60
50
40
30
20
0
50
100
150

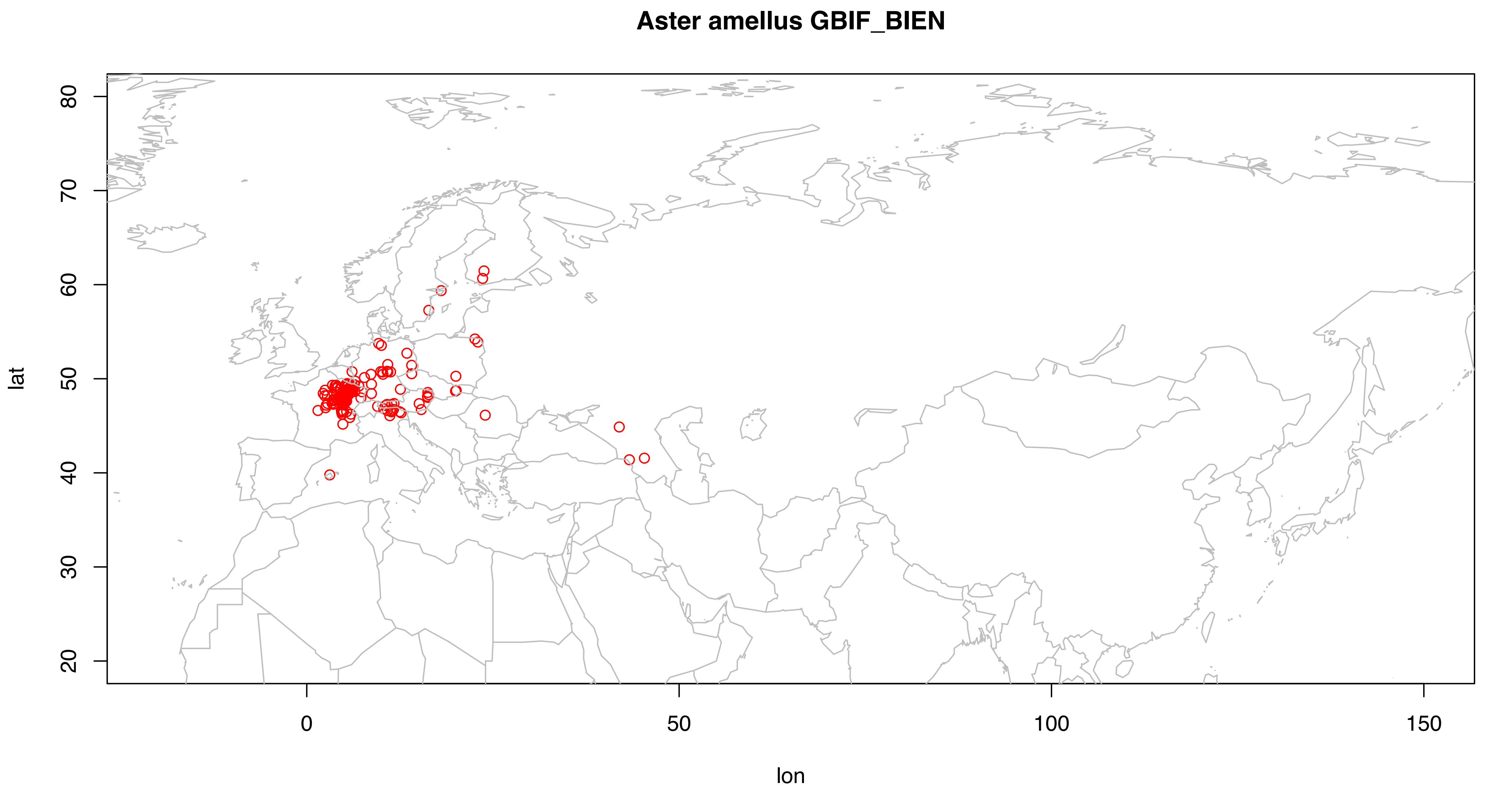
Aster amellus GBIF_BIEN
lat
lon
80
70
60
50
40
30
20
0
50
100
150

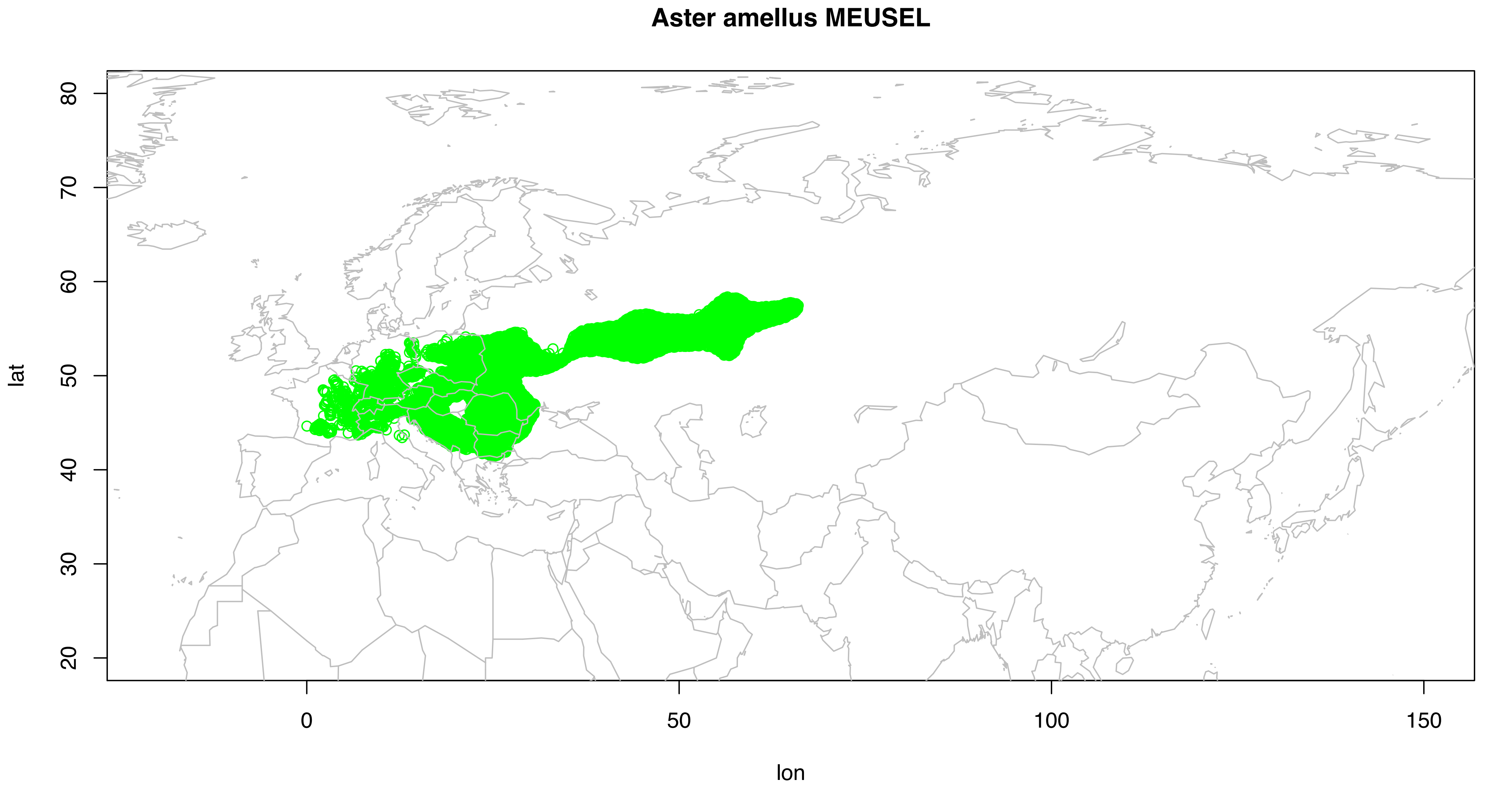
Aster amellus MEUSEL
lat
lon
80
70
60
50
40
30
20
0
50
100
150

Cirsium dissectum GBIF_BIEN

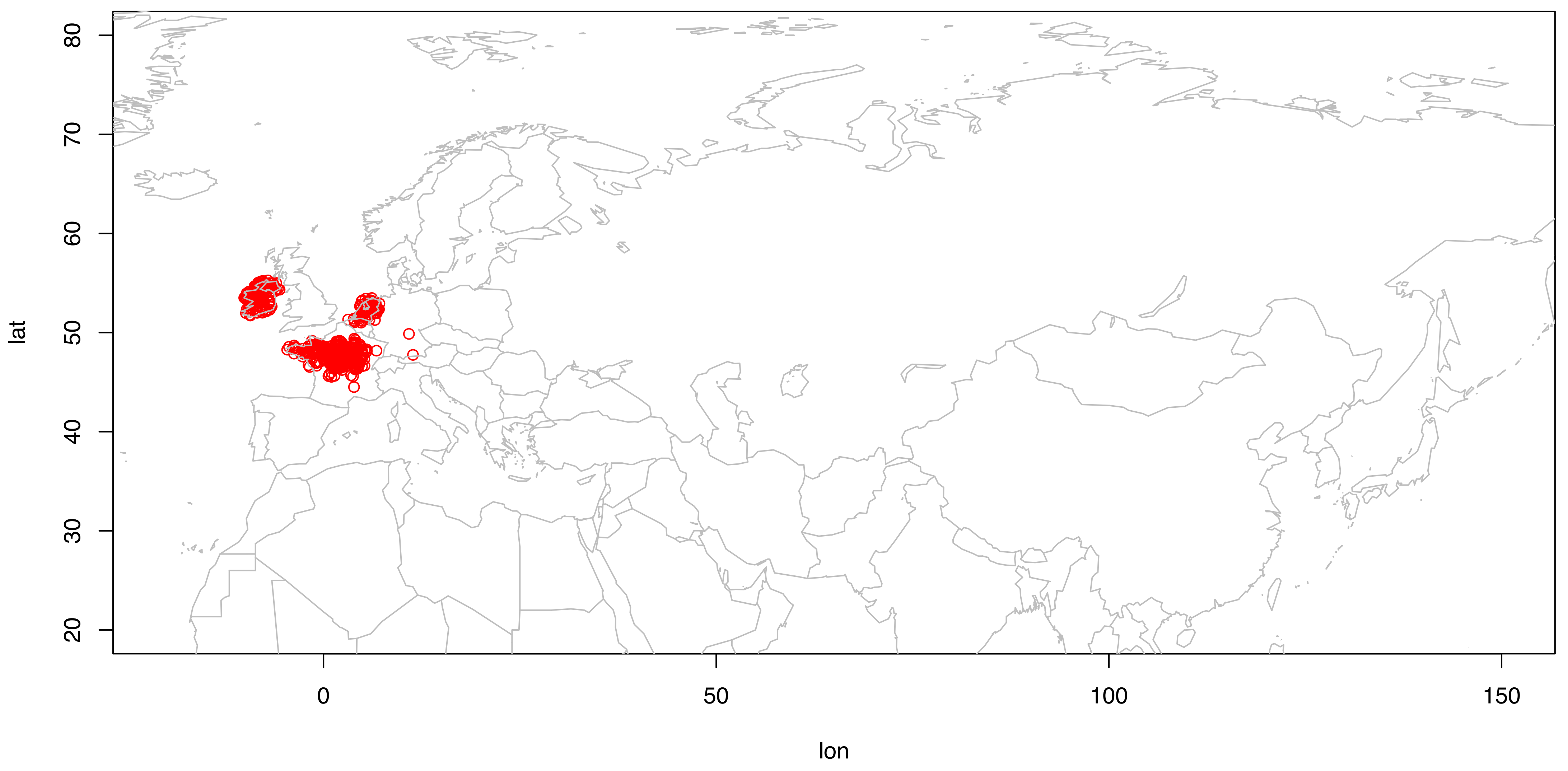

Cirsium dissectum MEUSEL

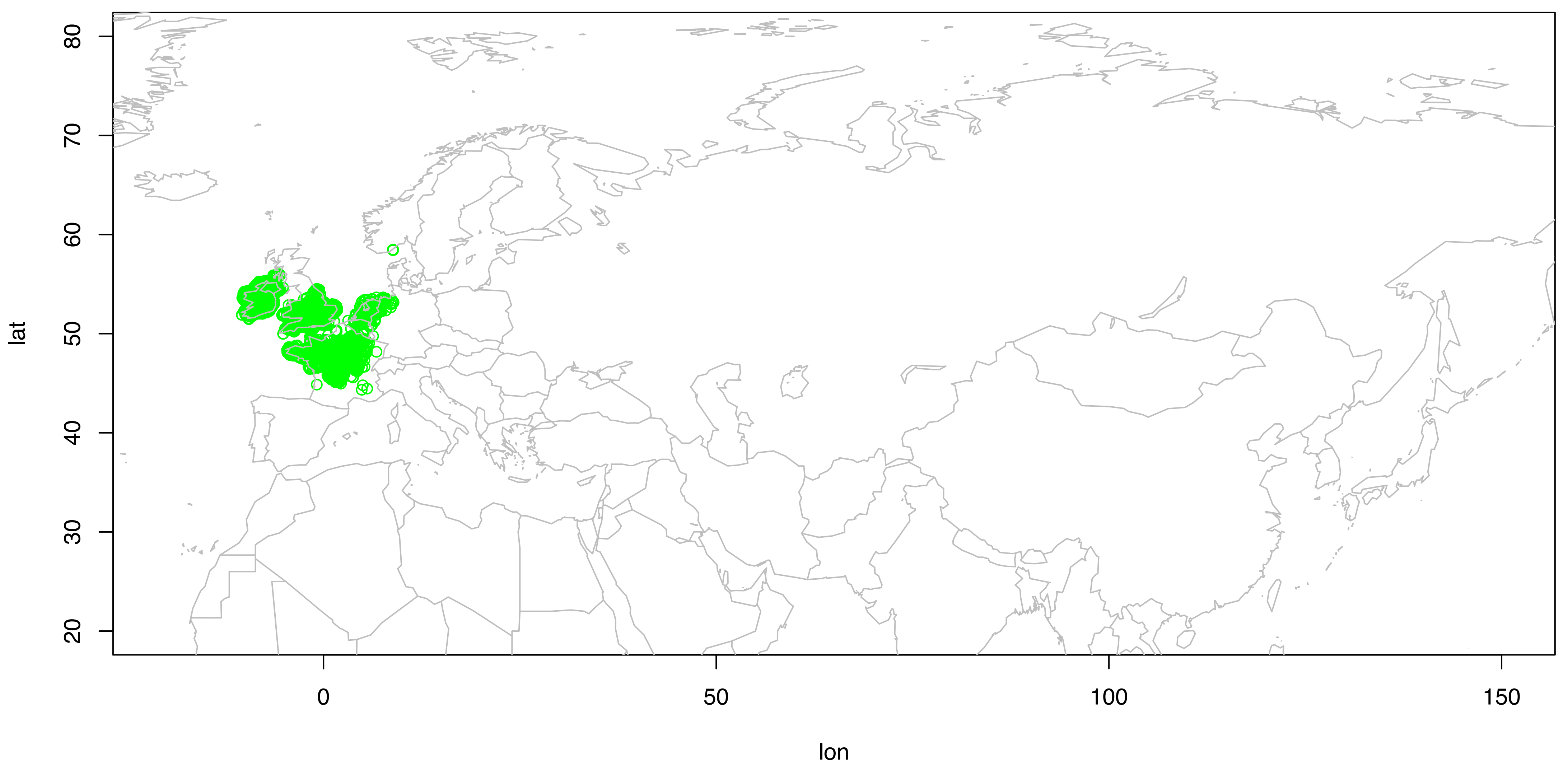

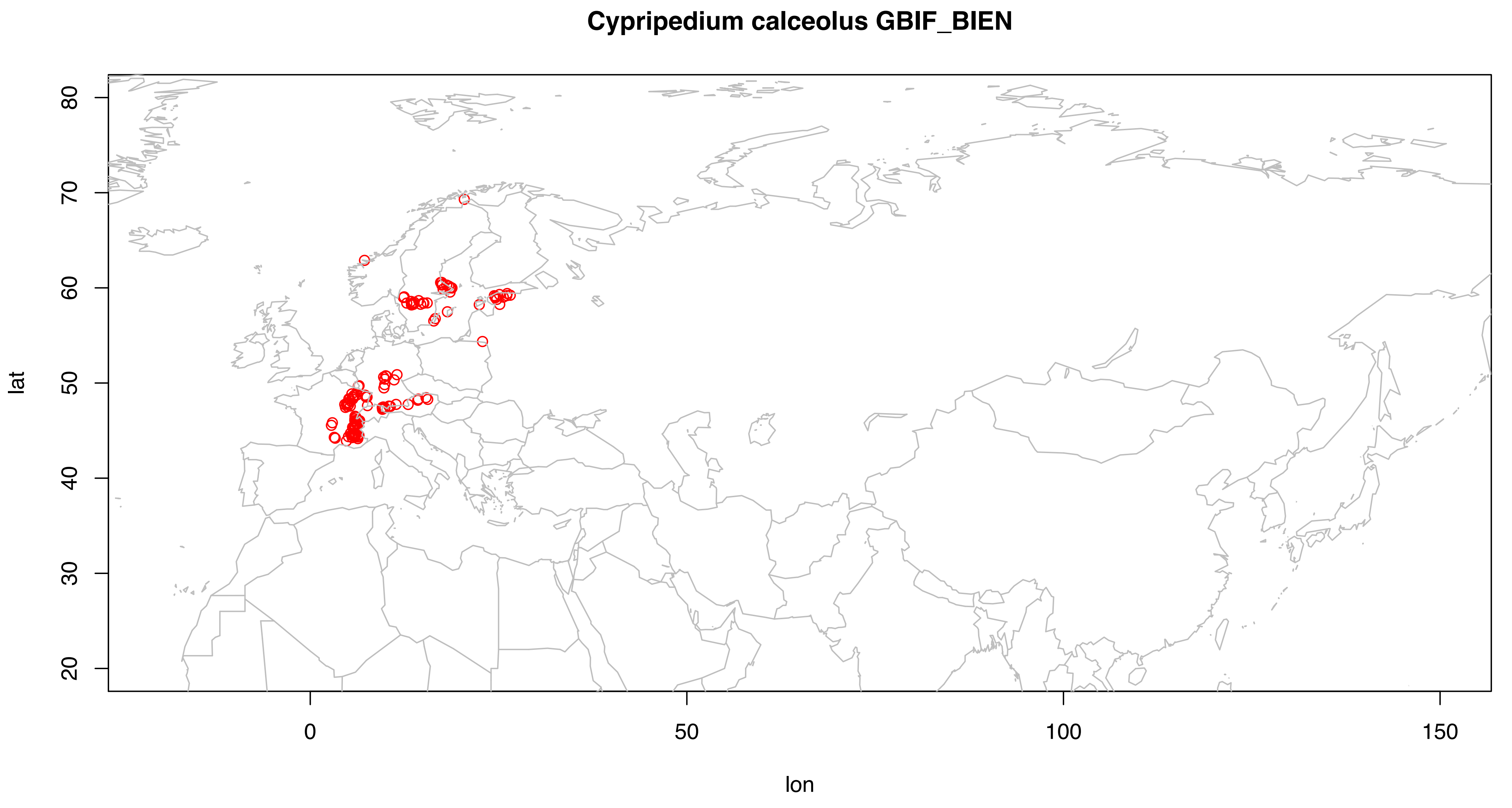
Cypripedium calceolus GBIF_BIEN
lat
lon
80
70
60
50
40
30
20
0
50
100
150

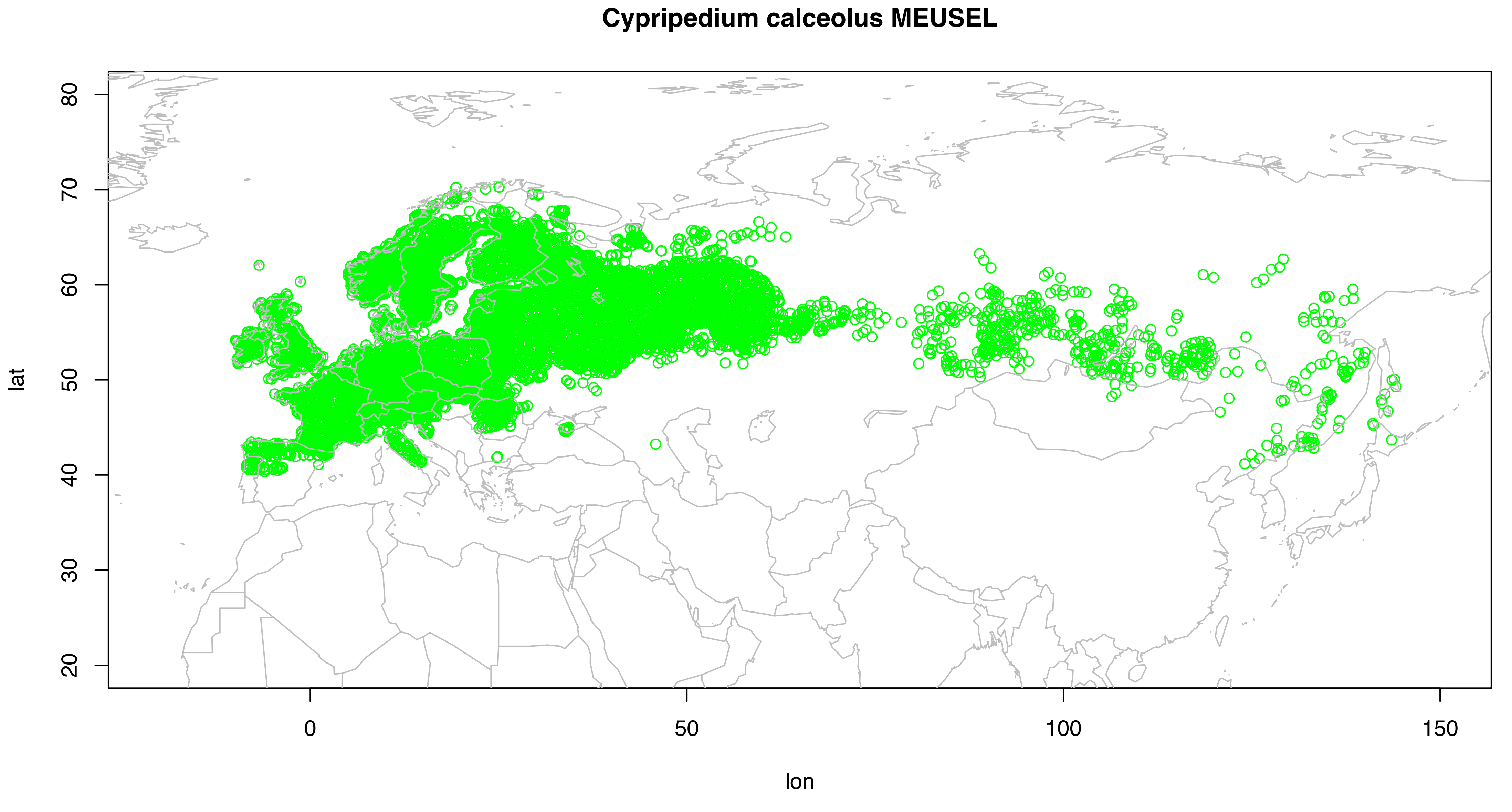
Cypripedium calceolus MEUSEL
lat
lon
80
70
60
50
40
30
20
0
50
100
150

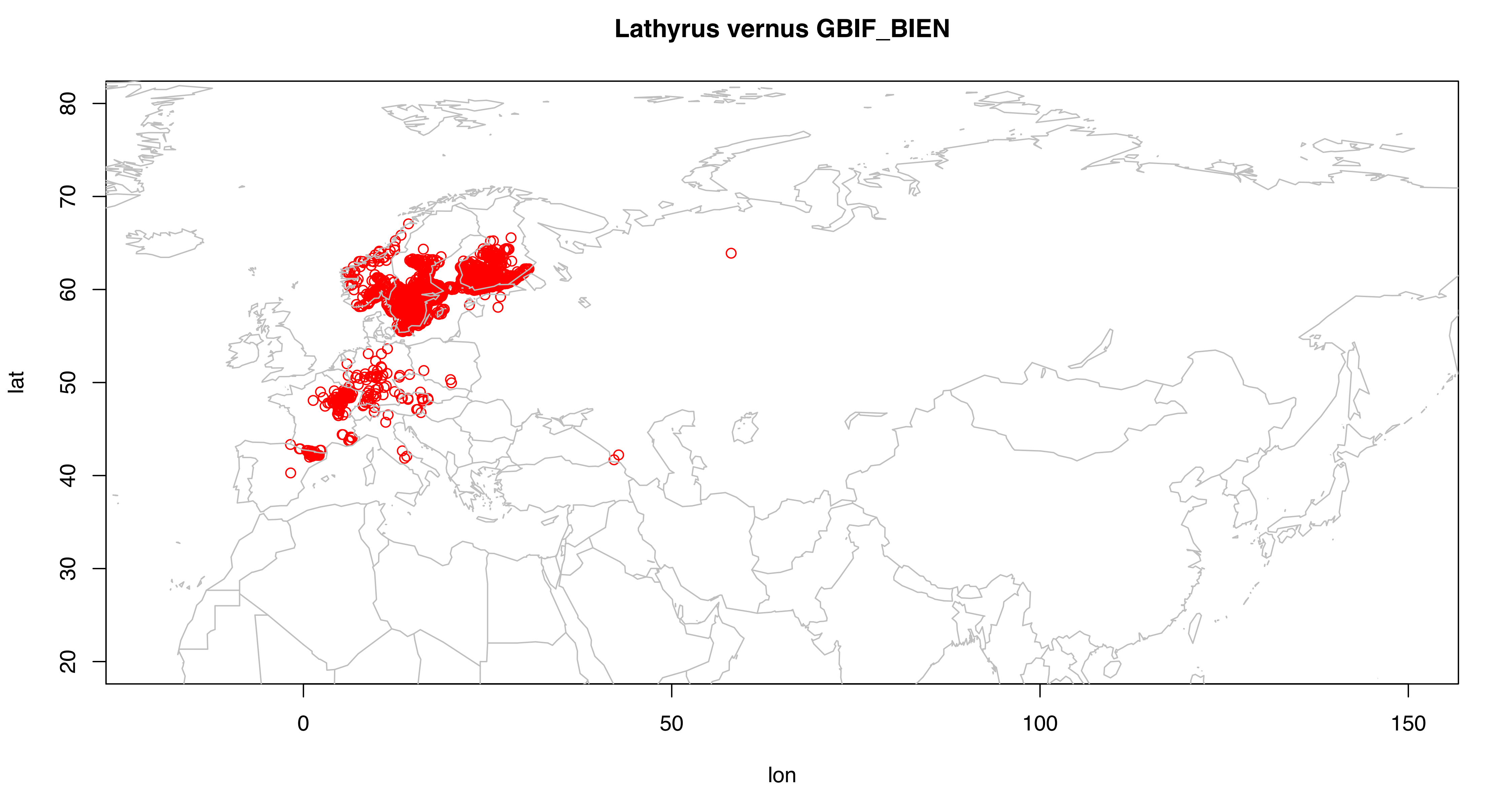
Lathyrus vernus GBIF_BIEN
lat
lon
80
70
60
50
40
30
20
0
50
100
150

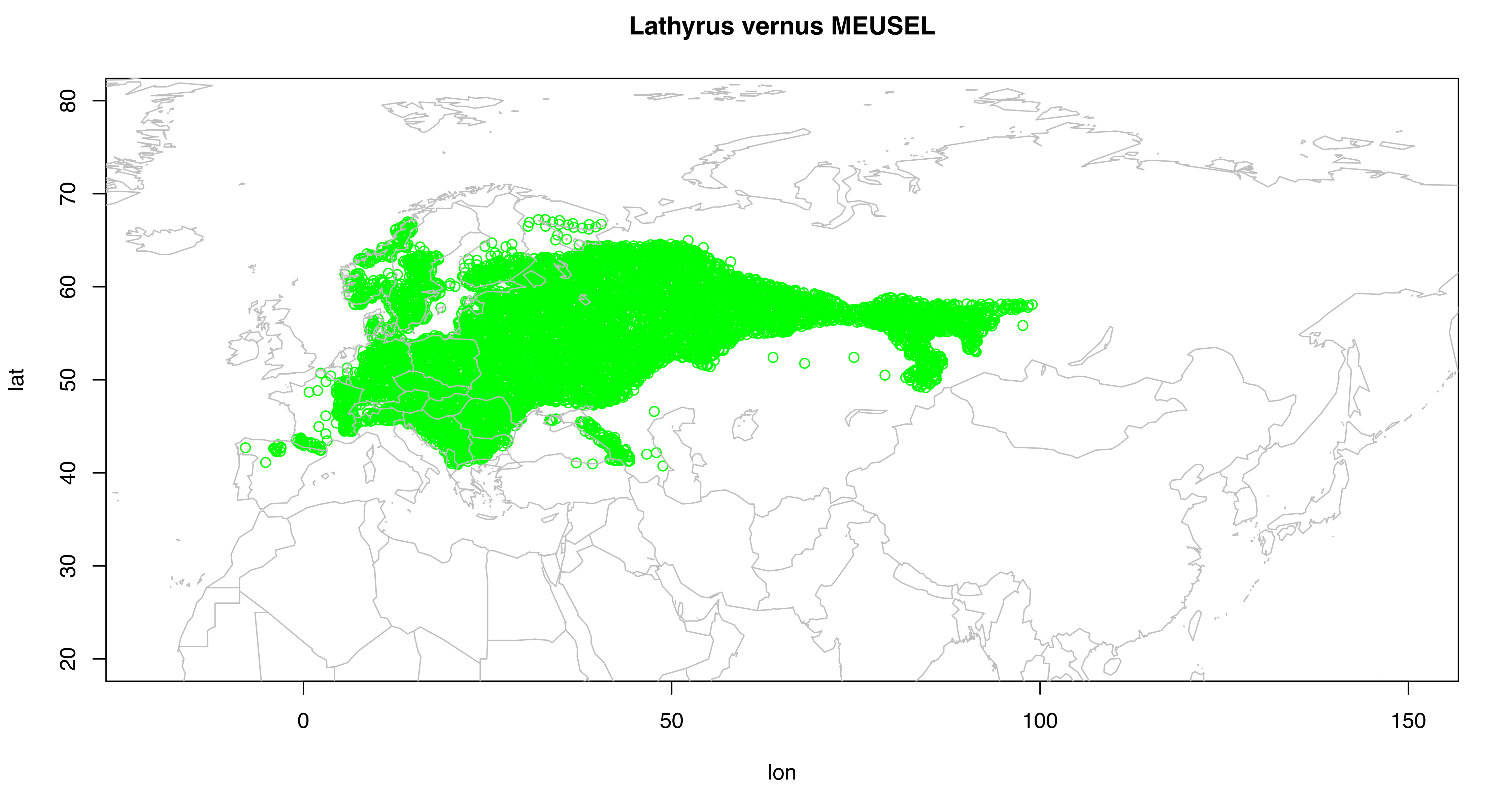
Lathyrus vernus MEUSEL
lat
lon
80
70
60
50
40
30
20
0
50
100
150

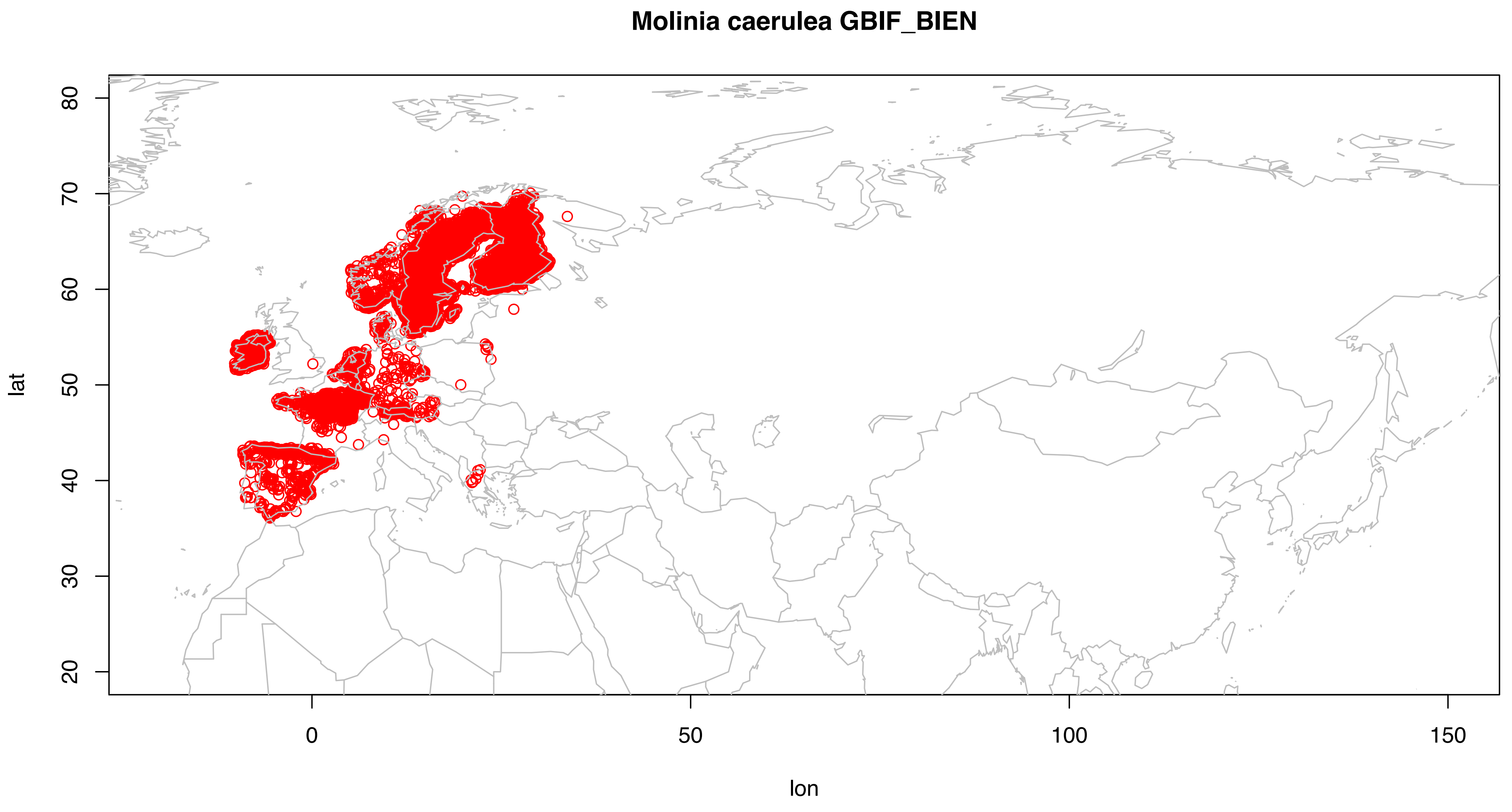

Molinia caerulea GBIF_BIEN
lat
lon
80
70
60
50
40
30
20
0
50
100
150

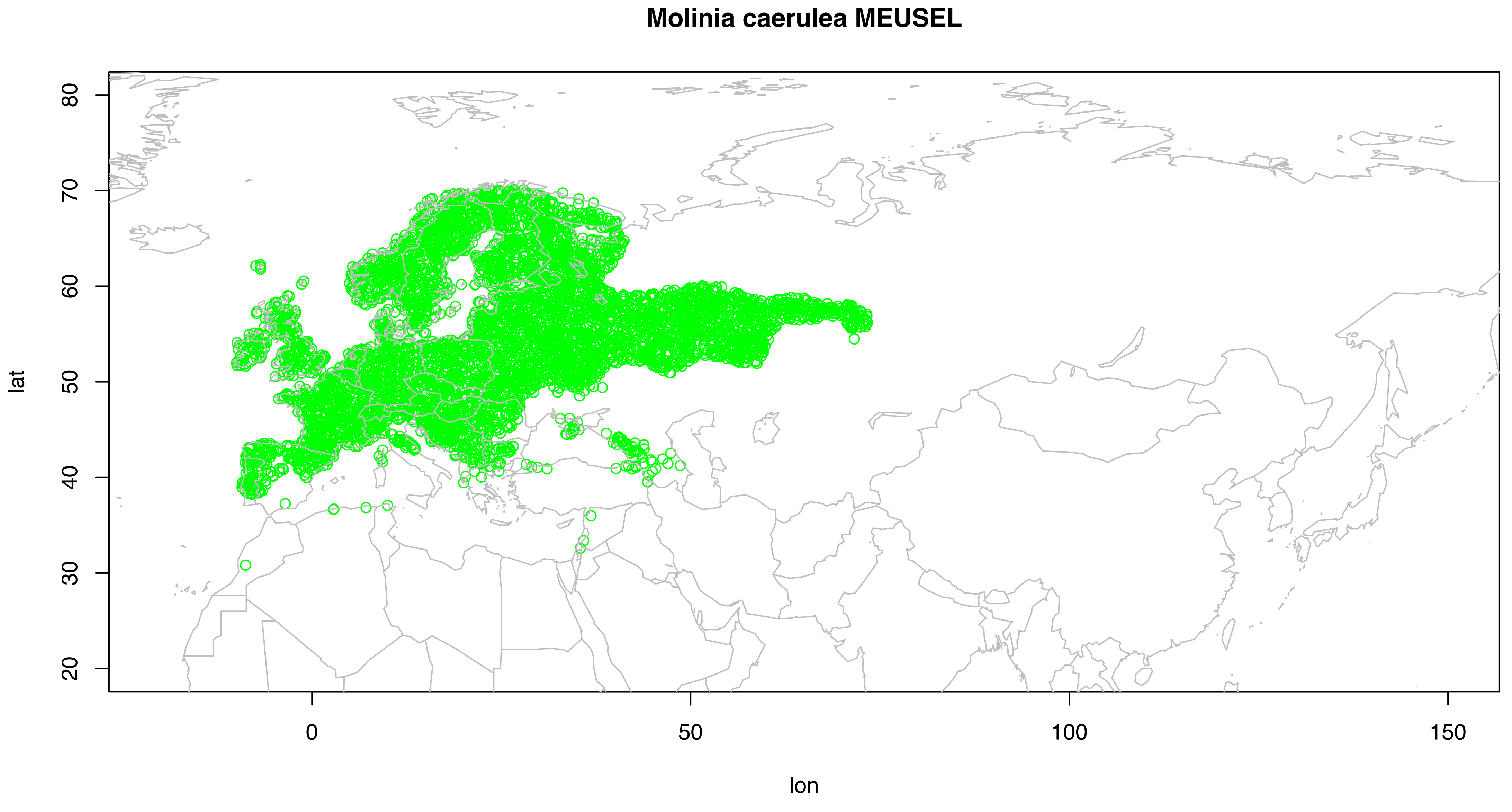

Molinia caerulea MEUSEL
lat
lon
80
70
60
50
40
30
20
0
50
100
150

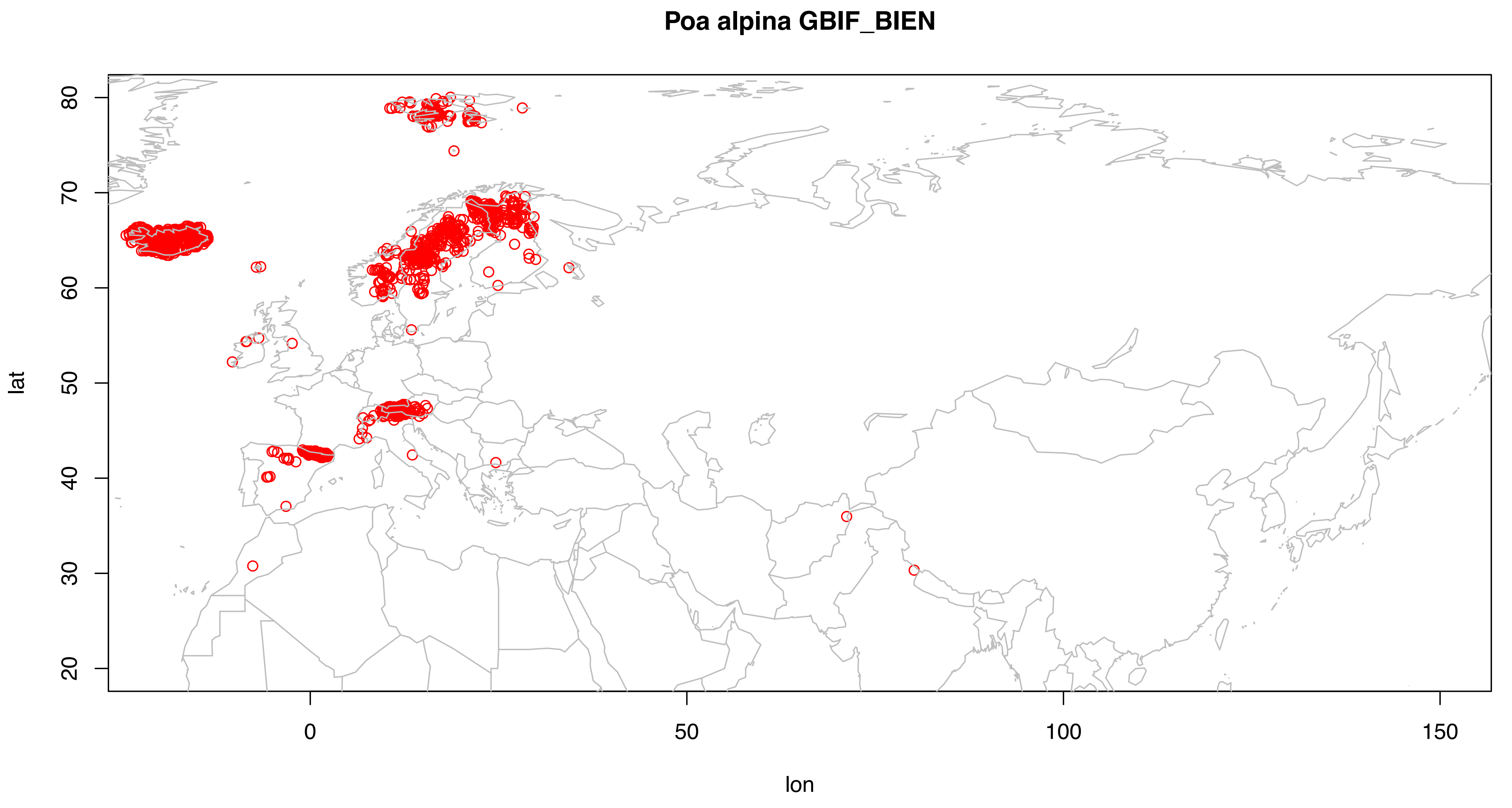

Poa alpina GBIF_BIEN
lat
lon
80
70
60
50
40
30
20
0
50
100
150

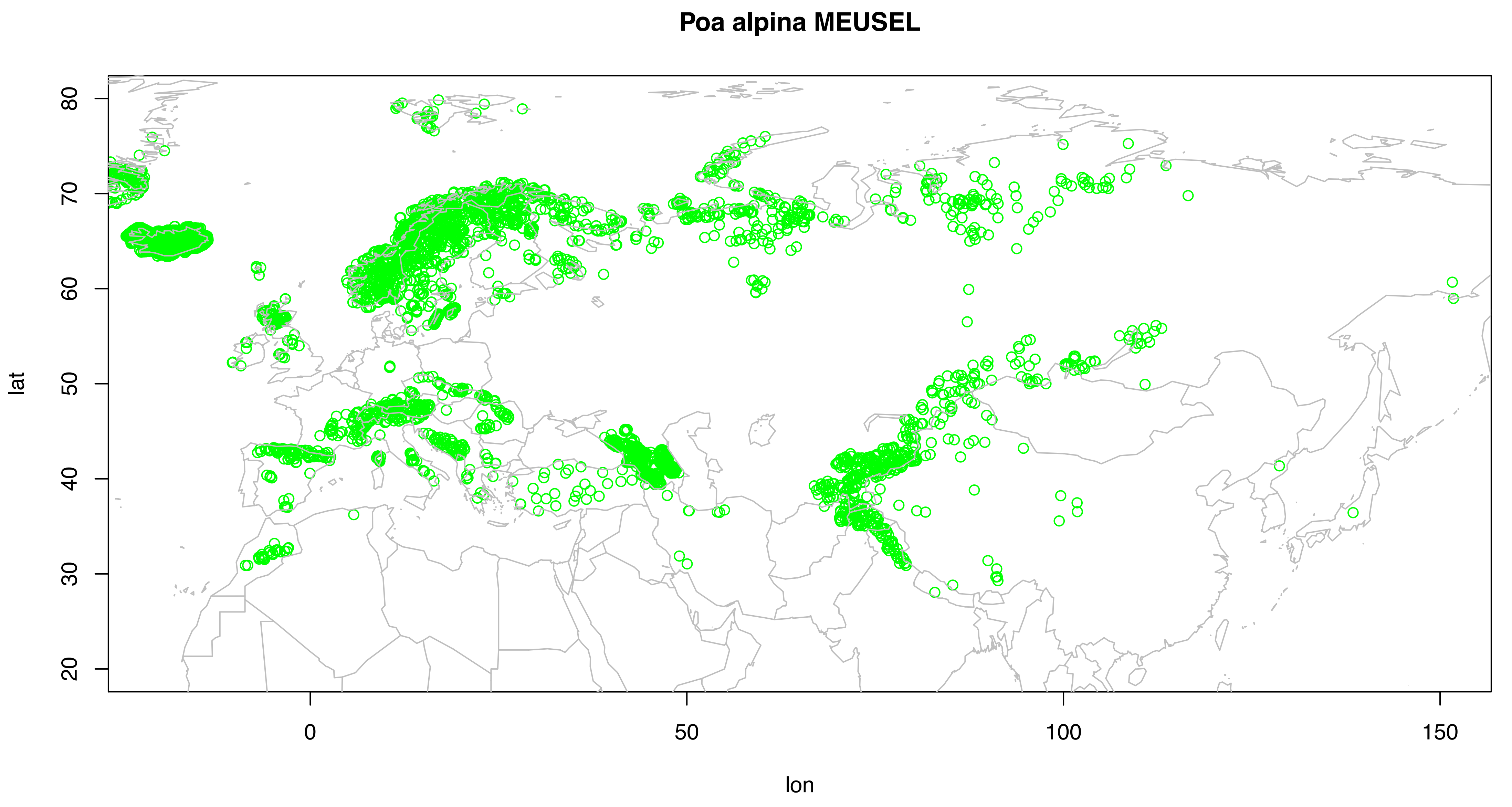

Poa alpina MEUSEL
lat
lon
80
70
60
50
40
30
20
0
50
100
150

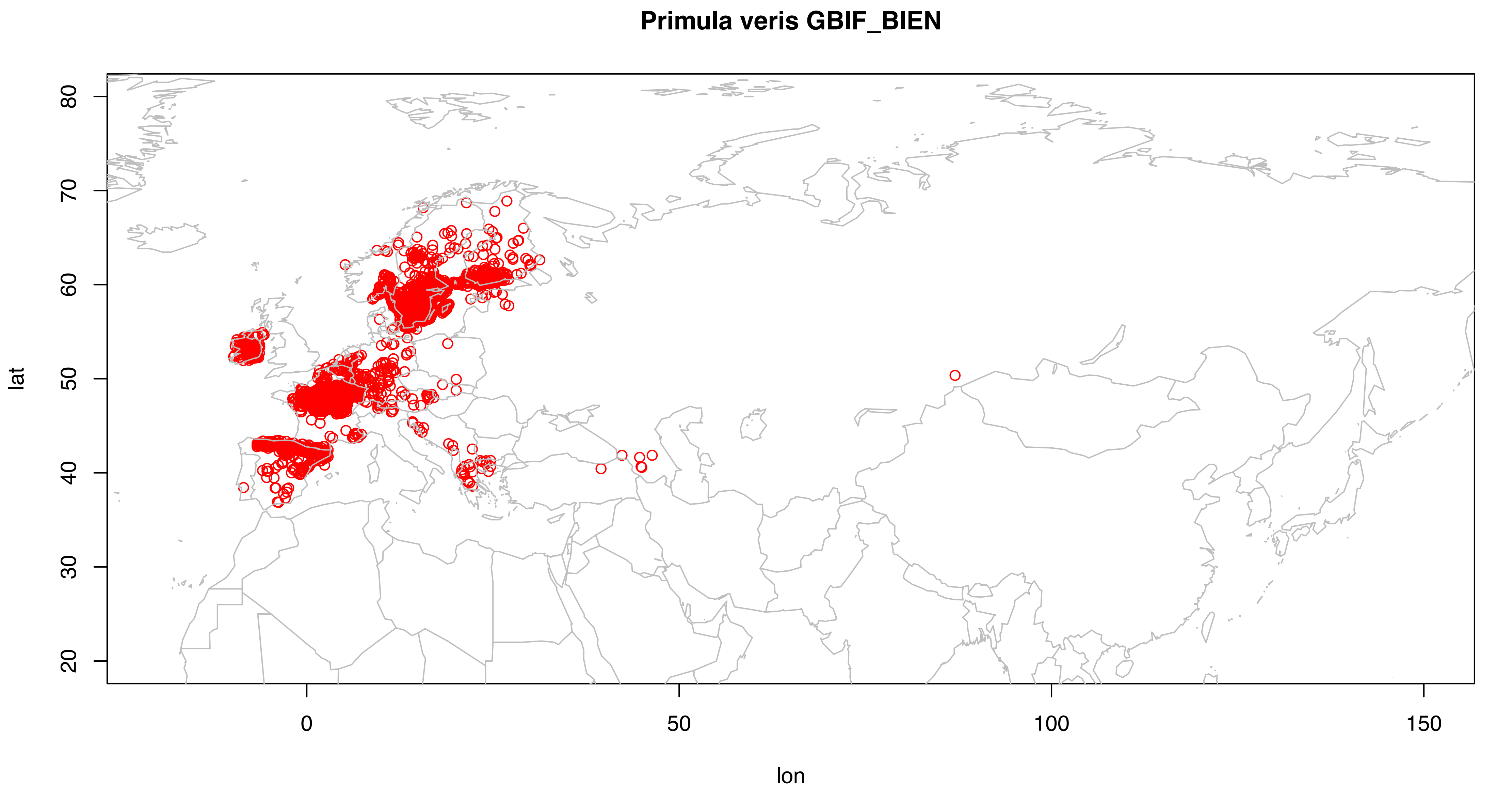
Primula veris GBIF_BIEN
lat
lon
80
70
60
50
40
30
20
0
50
100
150

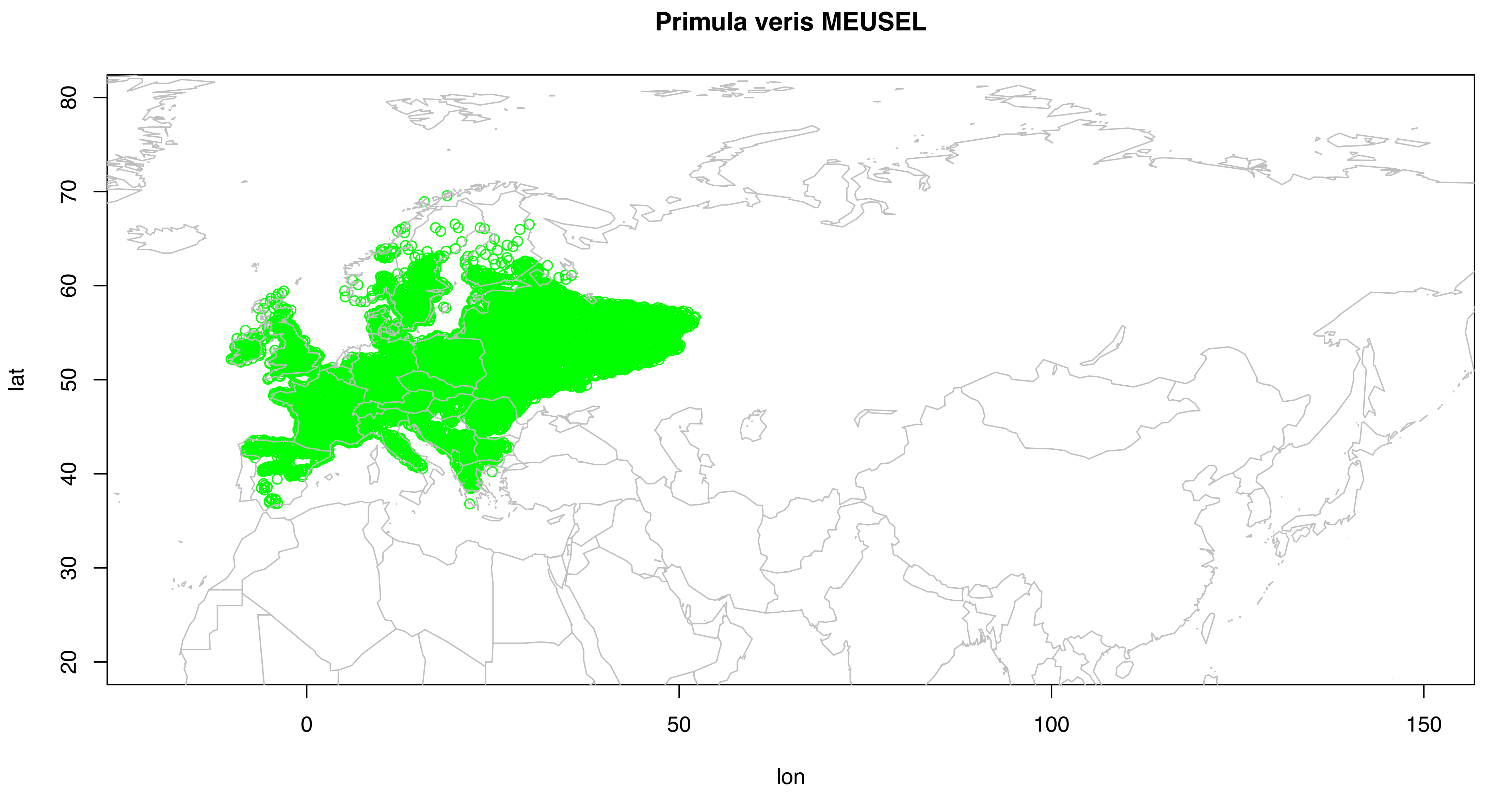
Primula veris MEUSEL
lat
lon
80
70
60
50
40
30
20
0
50
100
150

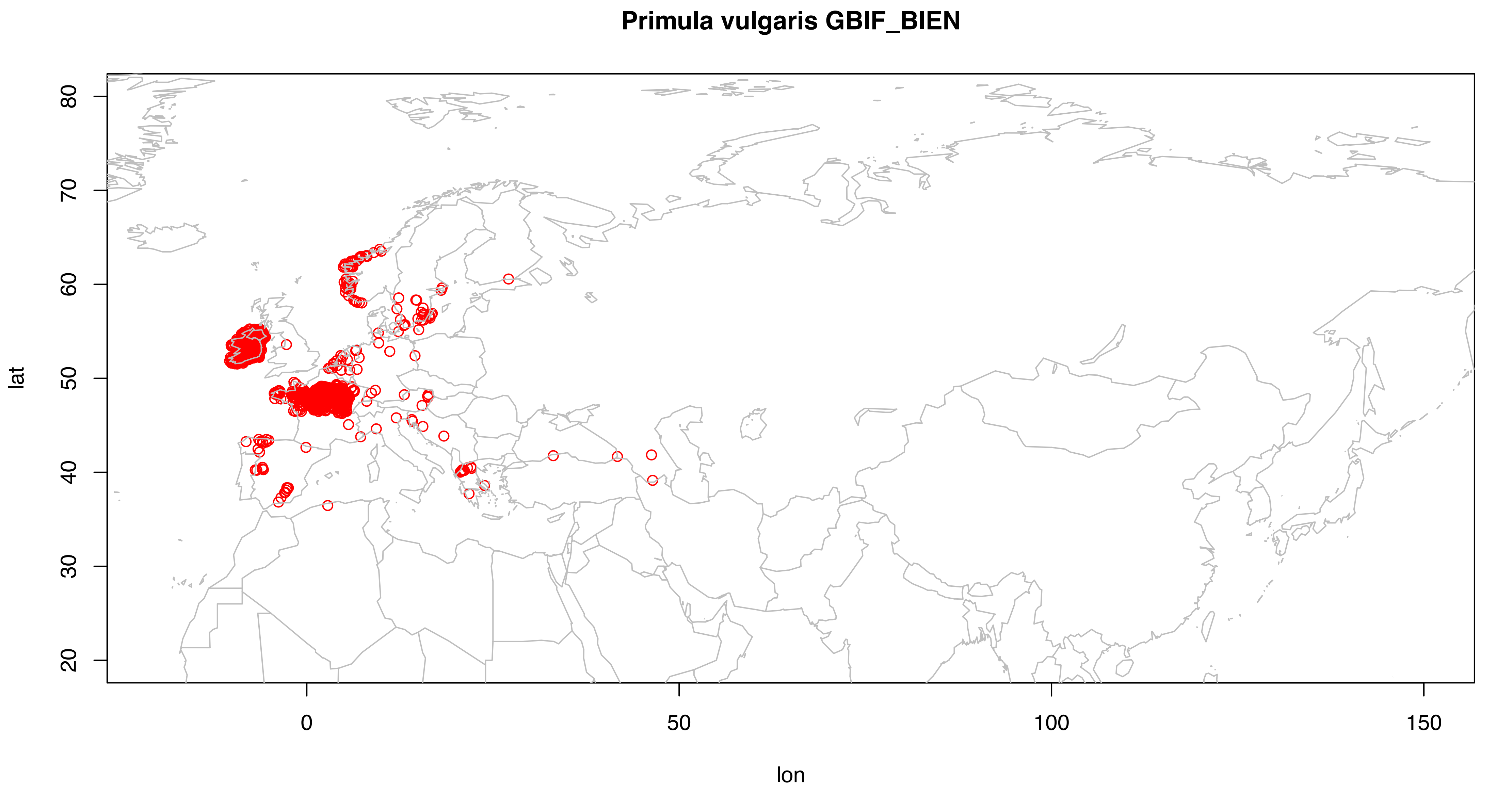

Primula vulgaris GBIF_BIEN
lat
lon
80
70
60
50
40
30
20
0
50
100
150

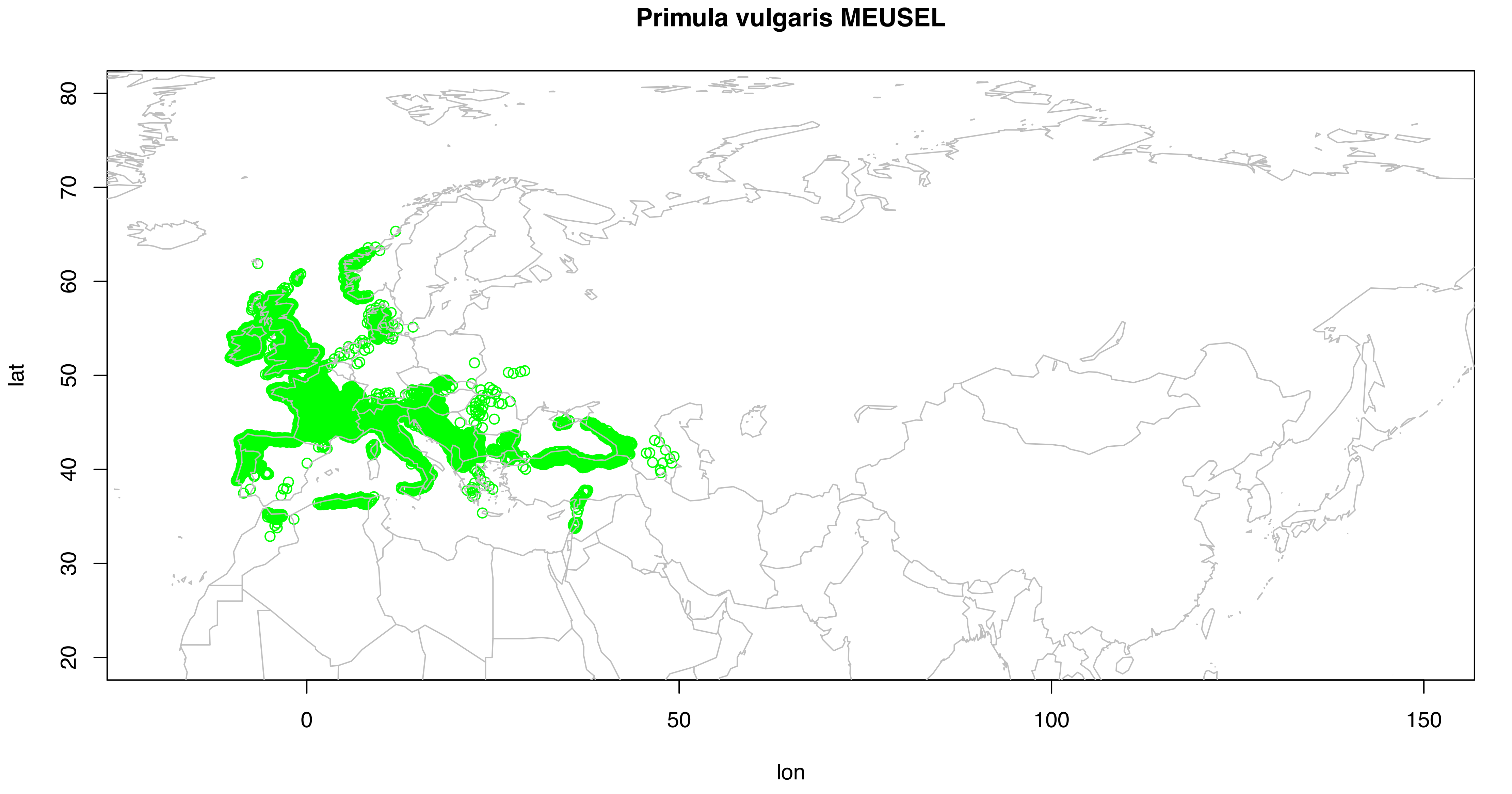

Primula vulgaris MEUSEL
lat
lon
80
70
60
50
40
30
20
0
50
100
150

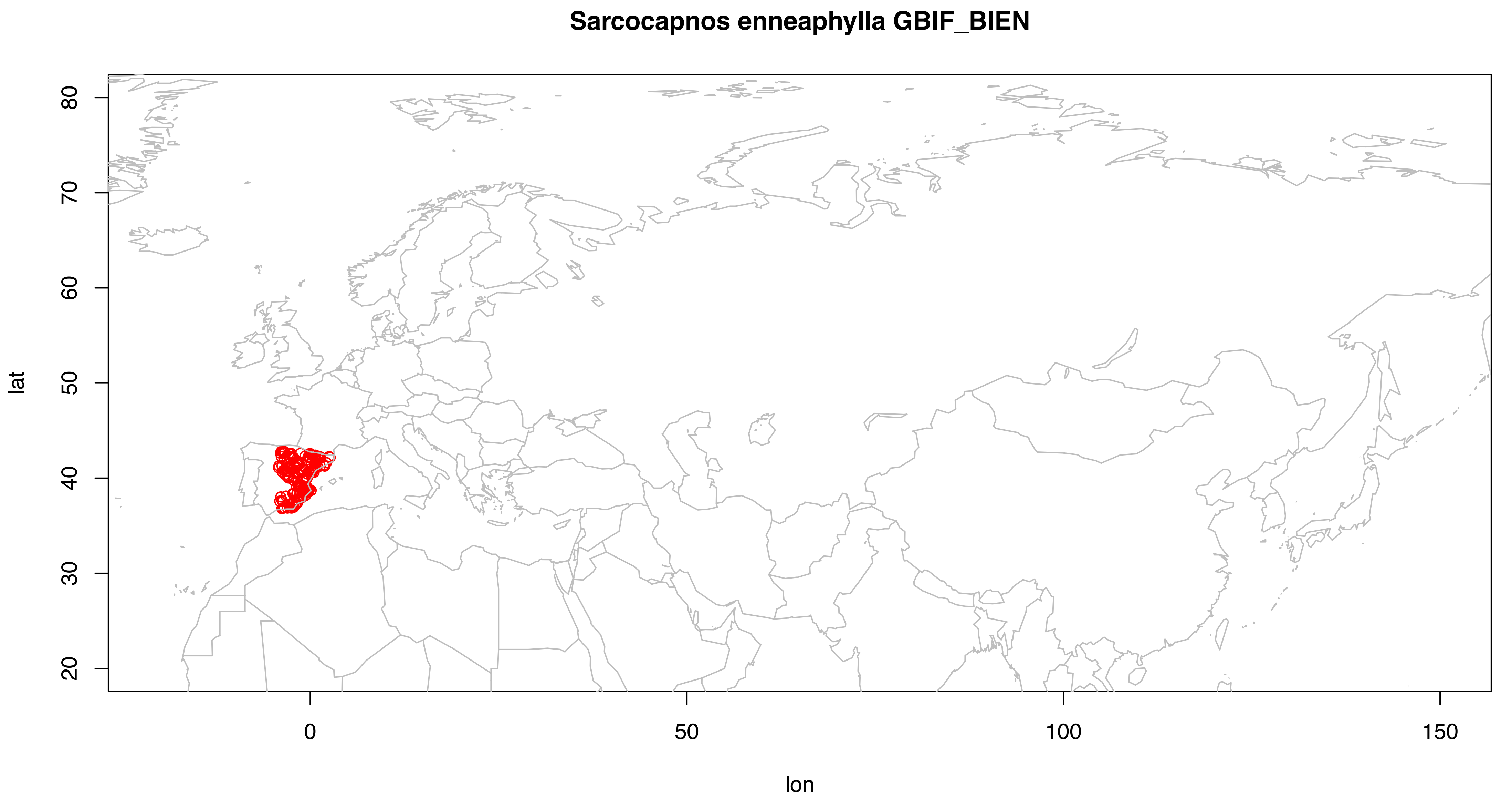
Sarcocapnos enneaphylla GBIF_BIEN
lat
lon
80
70
60
50
40
30
20
0
50
100
150

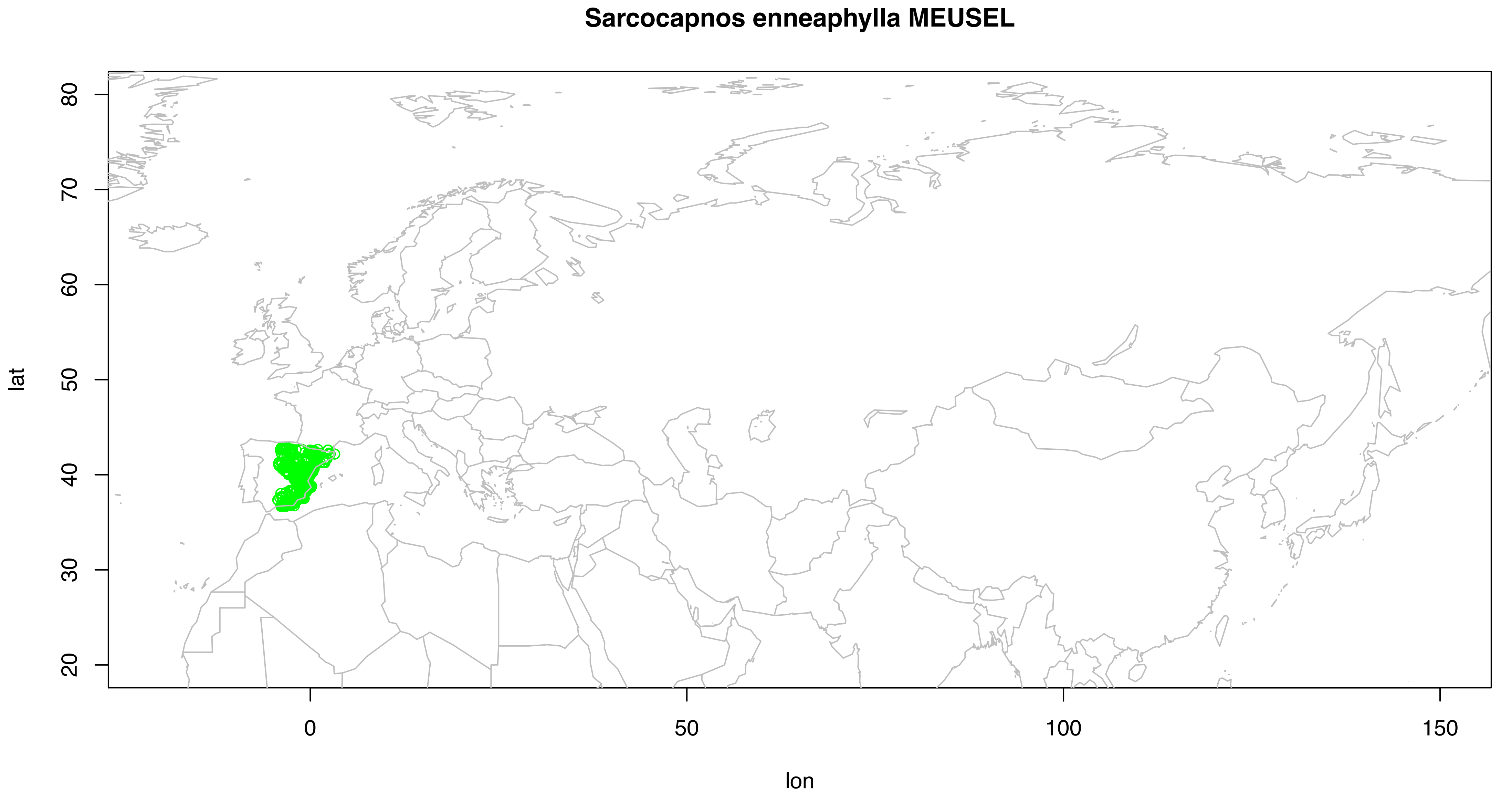
Sarcocapnos enneaphylla MEUSEL
lat
lon
80
70
60
50
40
30
20
0
50
100
150

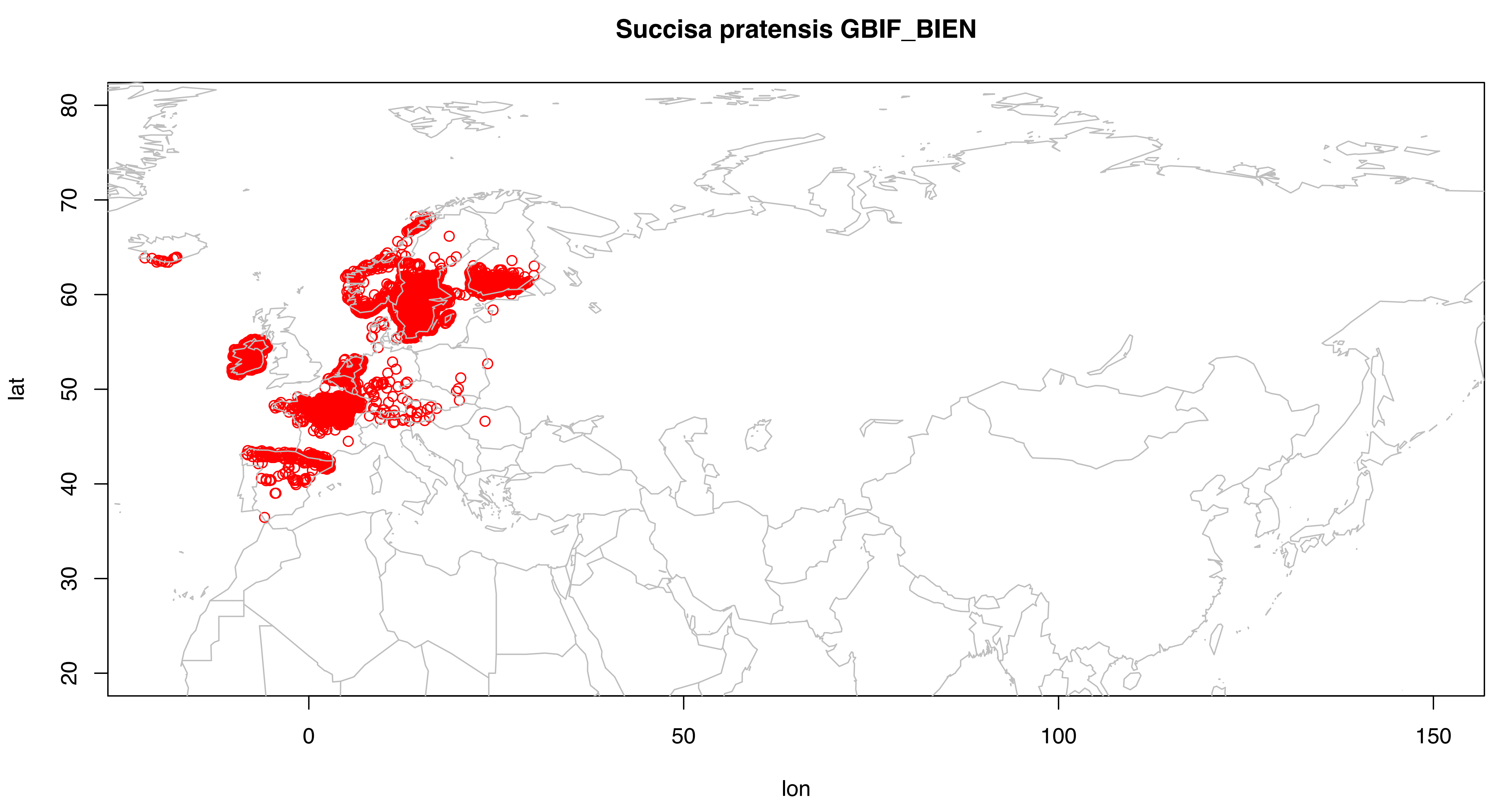
Succisa pratensis GBIF_BIEN
lat
lon
80
70
60
50
40
30
20
0
50
100
150

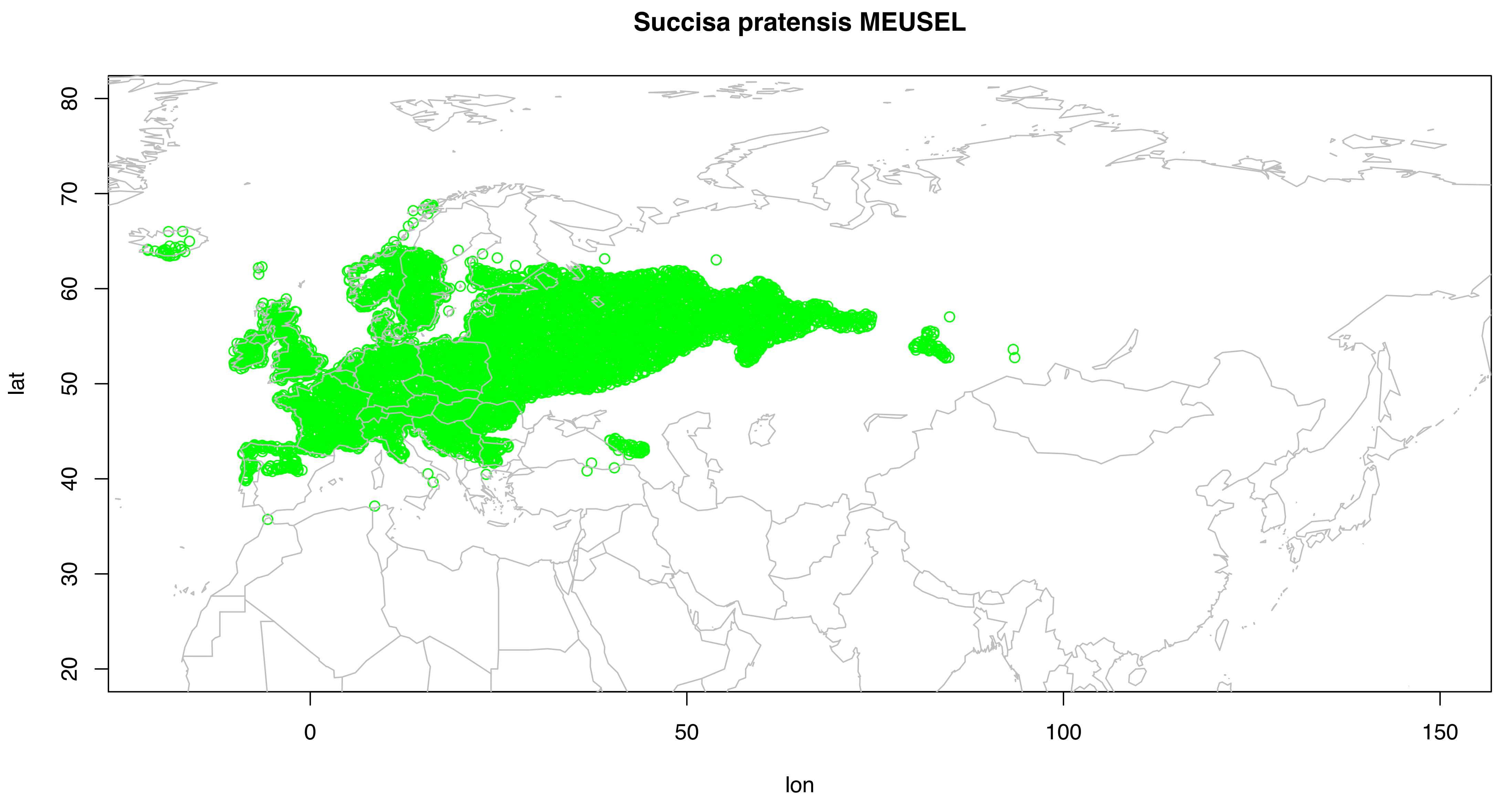
Succisa pratensis MEUSEL
lat
lon
80
70
60
50
40
30
20
0
50
100
150
