# Supplementary material for: Less favourable climates constrain demographic strategies in plants
Source: Ecol Lett. 2017 Jun 13;20(8):969–80. doi: 10.1111/ele.12794 (PMC5575490; doi:10.1111/ele.12794)

Supporting Information Appendix S2

Appendix S2.1 Description of demographic data

The final dataset contained 34 species with 93 populations, of which 13 species of trees with 29 populations and 21 species of herbaceous perennials with 64 populations. In this dataset, there were 2.7 ± 2.3 (mean±1SD) populations/species on average with a median of 2 populations, and 12 species had only one population. Populations were generally studied for a short time (3.0 ± 1.3 years – mean ± 1SD, with a median of 3 years), and projection matrices were built from 5.0 ± 1.3 (mean±1SD) developmental stages with a median of 5 stages.

The deterministic and stochastic population growth rates had very similar values $\boldsymbol{\lambda}_{Geom}$ = 1.03 ± 0.17 and $\boldsymbol{\lambda}_{iid}$ = 1.04 ± 0.20 (mean±1SD). Stasis was the dominant demographic process (0.120; 0.060 - 0.173) followed by progression (0.012; 0.005 - 0.024), fecundity (0.006; 0.003 - 0.015) and finally, retrogression (0.001; 0.000 - 0.007) (Median; 1st - 3rd Quartiles). The elasticity of population growth rates was highest for changes in stasis (0.666 ± 0.206; 0.668), followed by progression (0.206 ± 0.132; 0.204), retrogression (0.064 ± 0.064; 0.058) and finally fecundity (0.056 ± 0.070; 0.028) (mean ± 1SD; median).

Appendix S2.2

Figure S2.2a The position of stochastic population growth rates along the climate suitability axis for 93 populations across 34 species in COMPADRE Plant Matrix Database. Blue dots indicate trees, red dots indicate herbaceous perennials, and the zero-intercept lines represent stable (i.e., neither increasing nor declining) populations ($\log(\boldsymbol{\lambda}_{iid}) = 0$).

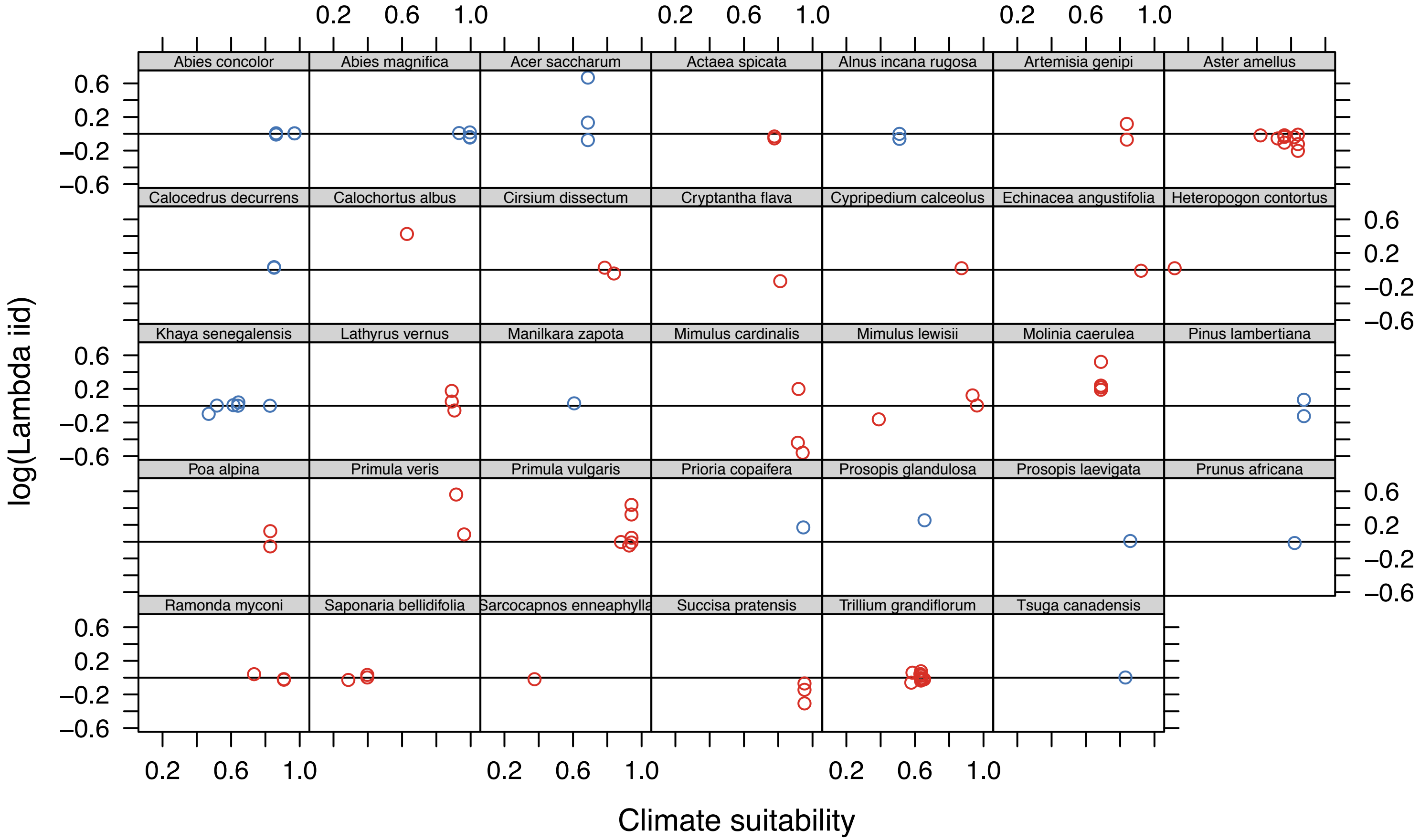

Figure S2.2b The position of projected extinction risk (time to quasi-extinction) along the climate suitability axis for 93 populations across 34 species in COMPADRE Plant Matrix Database. Blue dots indicate trees, red dots indicate herbaceous perennials. Simulations were stopped at 300 years.

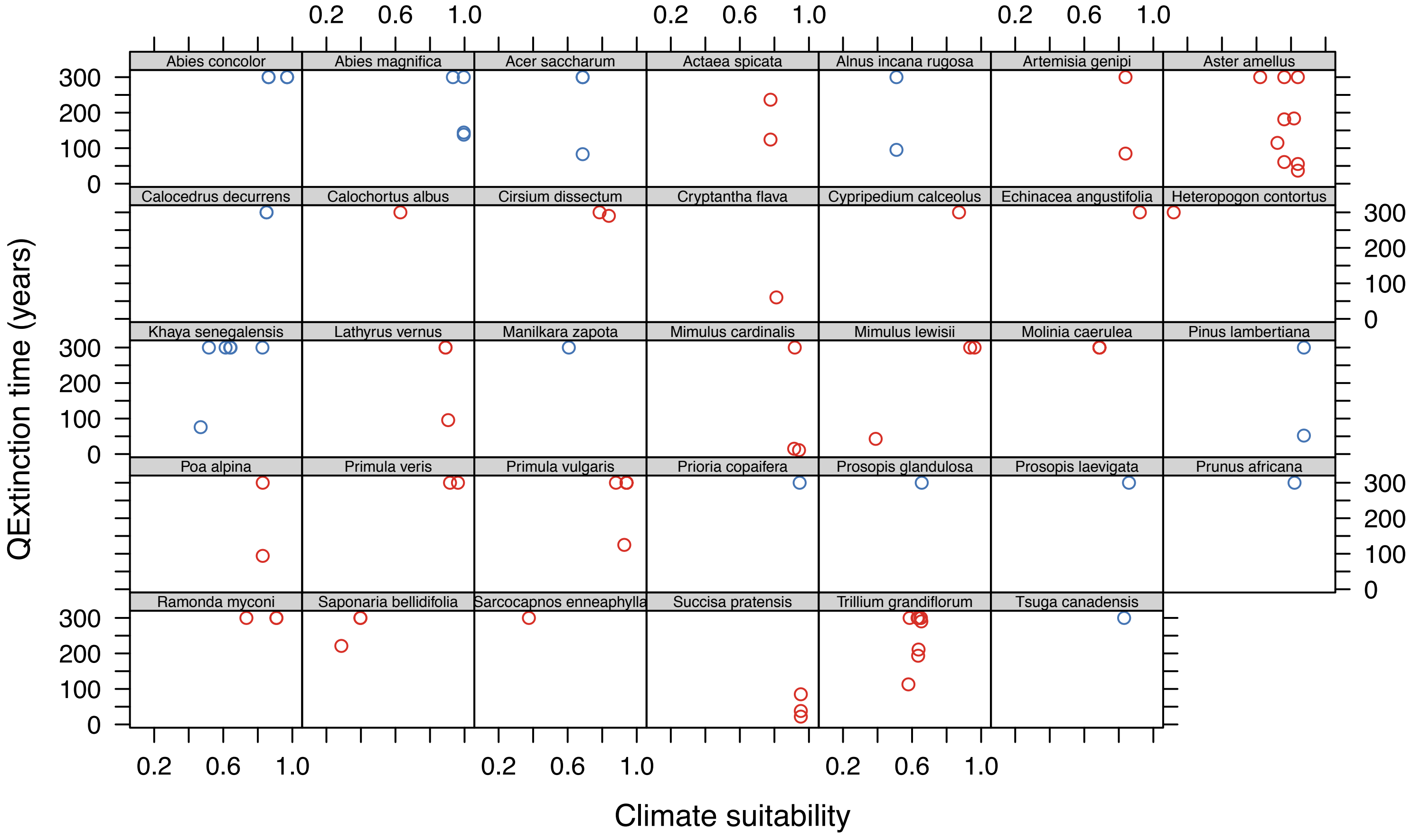

Appendix S2.3 (Table S2.3)

Best fit Linear Mixed Effects Models (LMMs) for the effects of climate suitability on population performance and the effects of demographic processes on population extinction risk. The first column shows the fixed effects in the full models and the abbreviated and full name of predicted variables. The next columns show the coefficient means β and standard errors SE(β) for variables selected in the best model, and marginal (fixed effects) R^2 values of the best models. In all models species (“SpeciesAccepted” column in COMPADRE) were introduced as random effects (intercept-only). MD = Matrix Dimension, SL = Study Length, GT = Growth Type, CS = Climate suitability, CV = Coefficient of Variation.

**Effects of climate suitability on mean and temporal variation of population growth rate, on extinction risk and transient dynamics**				
Model structure and predicted variable	Selected variable	β	SE(β)	R^2
log(λiid) ~ MD+SL+GT+CS+MD:CS+SL:CS+GT:CS				
Stochastic population growth rate	Intercept	0.972	0.031	0.000
log(CVλ_det) ~ MD+SL+GT+CS+MD:CS+SL:CS+GT:CS				
Temporal variation of deterministic population growth rates	Intercept	0.123	0.016	0.127
	GT Tree	-0.075	0.026	
log(timeto95ext_200) ~ MD+SL+GT+CS				
Time to 95% probability of quasi-extinction	Intercept	4.474	0.182	0.000
log(RR) ~ MD+SL+GT+CS+MD:CS+SL:CS+GT:CS				
Reactivity range	Intercept	2.751	0.541	0.029
	CS	0.555	0.235	
log(IR) ~ MD+SL+GT+CS+MD:CS+SL:CS+GT:CS				
Inertia range	Intercept	3.907	0.535	0.033
	CS	0.593	0.265	

Effects of climate suitability on mean and temporal variation of demographic processes

Model structure and predicted variable	Selected variable	β	SE(β)	R^2
log(fec) ~ MD+SL+GT+CS+MD:CS+SL:CS+GT:CS				
Fecundity	Intercept	-5.059	0.247	0.071
	MD	-0.415	0.211	
sqrt(progr) ~ MD+SL+GT+CS+MD:CS+SL:CS+GT:CS				
Progression	Intercept	0.108	0.010	0.059
	MD	-0.015	0.009	
stasis ~ MD+SL+GT+CS+MD:CS+SL:CS+GT:CS				
Stasis	Intercept	0.100	0.009	0.531
	GTTree	0.062	0.016	
	CS	-0.007	0.005	
	MD	-0.041	0.007	
log(retr) ~ MD+SL+CS+MD:CS+SL:CS				
Retrogression (Herbaceous perennials)	Intercept	-6.312	0.614	0.037
	CS	-0.540	0.219	
CV_fec ~ MD+SL+GT+CS+MD:CS+SL:CS+GT:CS				
Temporal variation of fecundity	Intercept	0.510	0.048	0.086
	CS	-0.116	0.044	
CV_prog ~ MD+SL+CS				
Temporal variation of progression (Trees)	Intercept	0.488	0.050	0.486
	CS	-0.253	0.055	
	MD	-0.088	0.044	
CV_prog ~ MD+SL+CS				
Temporal variation of progression (Herbaceous perennials)	Intercept	0.419	0.035	0.110
	MD	-0.103	0.037	
sqrt(CV_stasis) ~ MD+SL+GT+CS+MD:CS+SL:CS+GT:CS				
Temporal variation of stasis	Intercept	0.437	0.038	0.090
	GT Tree	-0.102	0.063	
	CS	-0.044	0.025	
log(CV_retr) ~ MD+SL+CS+MD:CS+SL:CS				
Temporal variation of retrogression (Herbaceous perennials)	Intercept	-0.817	0.100	0.044
	SL	0.158	0.094	

Effects of mean and temporal variation of demographic processes on population extinction risk

Model structure and predicted variable	Selected variable	β	SE(β)	R^2
timeto95ext_200~ MD+SL+Fec+Sta+Ret+Prog				
Time to 95% probability of quasi-extinction	Intercept	116.909	14.236	0.409
	MD	34.433	16.206	
	Retrogression	54.431	15.368	
	SL	23.596	13.048	
timeto95ext_200~ MD+SL+CV_fec+CV_Sta+CV_Ret+CV_Prog				
Time to 95% probability of quasi-extinction	Intercept	113.42	14.35	0.000
	CV_progression	-21.99	10.79	
	CV_Stasis	20.70	12.11	
	SL	29.76	13.68	

Effects of climate suitability on the elasticity of population growth rate to changes in demographic processes

Model structure and predicted variable	Selected variable	β	SE(β)	R^2
*ElastFec ~ MD+SL+GT+CS+λiid+MD:CS+SL:CS+GT:CS+*λiid:CS				
Elasticity of *λ* to changes in mean fecundity	Intercept	0.077	0.011	0.401
	GT Tree	-0.042	0.018	
	CS	-0.018	0.005	
	λiid	0.032	0.003	
	SL	-0.004	0.005	
	GTTree:CS	0.029	0.011	
	λiid:CS	0.012	0.004	
	SL:CS	0.016	0.006	
ElastProg ~ MD+SL+GT+CS+λiid+MD:CS+SL:CS+GT:CS+λiid:CS				
Elasticity of *λ* to changes in mean progression	Intercept	0.251	0.021	0.506
	GT Tree	-0.138	0.035	
	CS	-0.007	0.013	
	λiid	0.066	0.007	
	MD	0.042	0.016	
	CS:MD	0.023	0.013	
ElastStasis ~ MD+SL+GT+CS+λiid+MD:CS+SL:CS+GT:CS+λiid:CS				
Elasticity of *λ* to changes in mean stasis	Intercept	0.564	0.033	0.420
	GT Tree	0.292	0.054	
	CS	0.015	0.019	
	λiid	-0.082	0.010	
	MD	-0.062	0.024	
	CS:MD	-0.037	0.020	
ElastRetr ~ MD+SL+GT+CS+λiid+MD:CS+SL:CS+GT:CS+λiid:CS				
Elasticity of *λ* to changes in mean retrogression	Intercept	0.092	0.013	0.320
	GT Tree	-0.064	0.031	
	CS	-0.005	0.006	
	λiid	-0.030	0.005	
	SL	0.008	0.007	
	CS:SL	-0.020	0.008	

Appendix S2.4

Figure S2.4 The relationship between the elasticity of population growth rate to basic demographic processes and climate suitability in interaction with other factors detected by the Linear Mixed Effects Models detailed in Appendix S2.3. Red lines represent minimum, orange lines represent median and blue lines represent maximum values of the factor in interaction with climate suitability. Dots represent 93 populations across 34 species of trees and herbaceous perennials. Black dots represents trees, and grey dots represent herbaceous perennials. Axis x represents climate suitability values centered on 0, with unit variance.

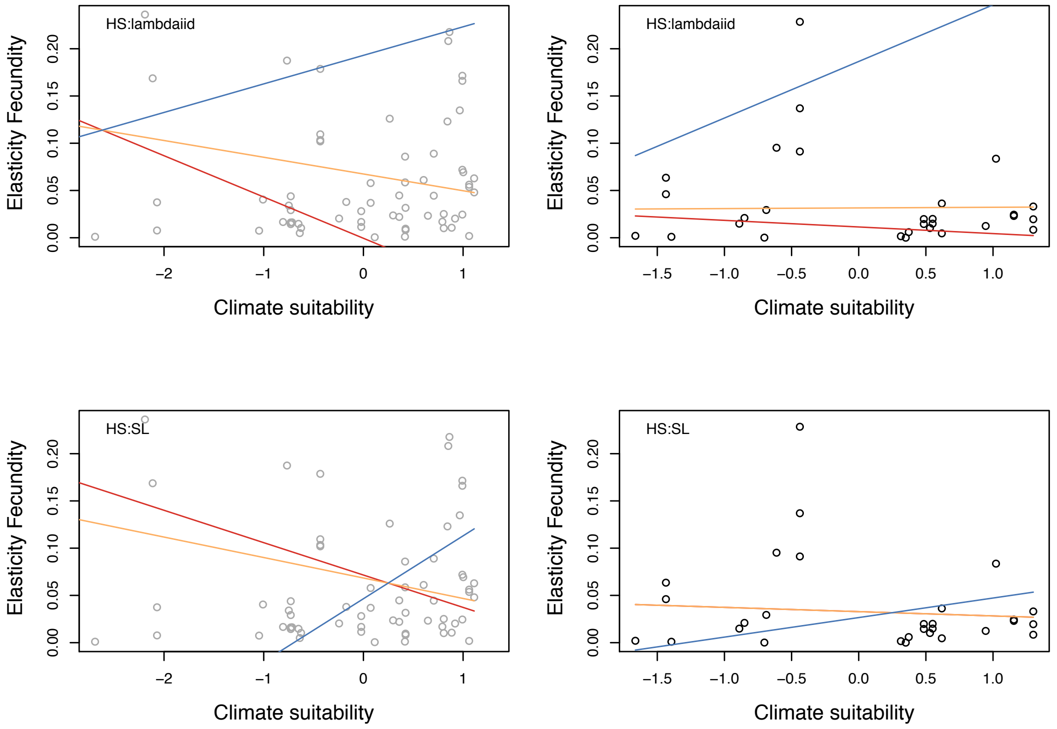

Figure S2.4 (continued)

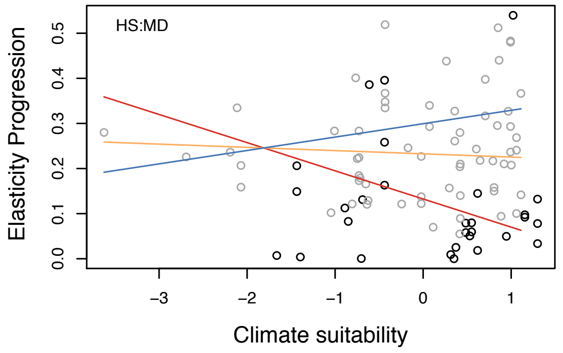

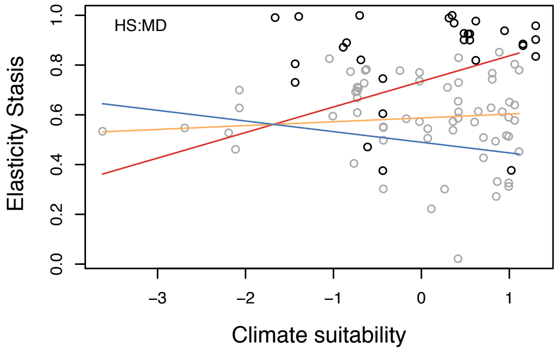

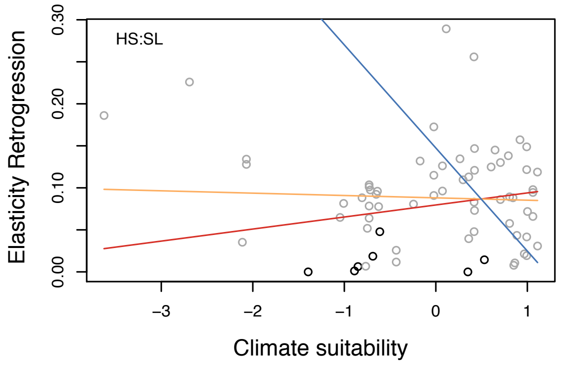

Supplement: Supplementary file 2 [file ELE-20-969-s002.pdf]
